# Extracting *Ligusticum chuanxiong* Hort. cultivation plots based on feature variable combinations constructed from UAV-based RGB images

**DOI:** 10.3389/fpls.2025.1659442

**Published:** 2025-11-10

**Authors:** Shihong Zhong, Rui Gu, Rong Ding, Yu Liang, Guihua Jiang, Chenghui Wang

**Affiliations:** 1 School of Pharmacy, Southwest Minzu University, Chengdu, China; 2 School of Pharmacy, Chengdu University of Traditional Chinese Medicine, Chengdu, China; 3 State Key Laboratory of Southwestern Chinese Medicine Resources, School of Pharmacy, Chengdu University of Traditional Chinese Medicine, Chengdu, China; 4 School of Ethnic Medicine, Chengdu University of Traditional Chinese Medicine, Chengdu, China

**Keywords:** *Ligusticum chuanxiong* Hort., unmanned aerial vehicle (UAV), machine learning, remote sensing, plot extraction

## Abstract

**Introduction:**

Accurate plots distribution mapping of the renowned Chinese medicinal plant, *Ligusticum chuanxiong* Hort. (LC) is crucial for its field management and yield estimation. However, due to the high fragmentation of LC cultivation plots, accurate classification using UAV-based RGB remote sensing images is challenging.

**Methods:**

This study utilized unmanned aerial vehicle RGB images to investigate the high-precision extraction of LC cultivation plots based on feature variable combinations across four representative sites: Site 1 (S1, traditional LC cultivation area in Dujiangyan City), Site 2 (S2, concentrated LC plots in Dujiangyan City), Site 3 (S3, traditional LC cultivation area in Pengzhou City), and Site 4 (S4, newly-developed LC cultivation area in Mianzhu City). Initially, appropriate color indices, texture features, color spaces, and digital elevation models were extracted from RGB images to form feature variable combinations. Subsequently, pixel-based classification and object-oriented classification methods were employed to construct LC cultivation plot extraction models.

**Results:**

The results showed that compared with classification results based on RGB images, the object-oriented classification method (k-nearest neighbor, KNN) based on feature variable combinations showed the highest overall classification accuracy and Kappa coefficient. The average Kappa coefficients for the classification of S1, S2, S3, and S4 were 0.86, 0.94, 0.93, and 0.90, respectively, while the overall accuracy rates were 89.16%, 95.72%, 94.55%, and 92.25%, respectively. The F1 scores averaged 99.62%, 98.11%, 96.11%, and 97.75%, respectively. Across all four sites, the mean Kappa coefficient, overall accuracy, and F1 score were 0.92, 92.92%, and 97.90%, respectively, showing an increase of 0.14, 14.17%, and 4.9% compared to the RGB images.

**Conclusions:**

The results indicate that the feature variable combination constructed based on UAV-based RGB remote sensing images can enhance the extraction accuracy of LC’s cultivation plots without incurring additional data acquisition costs. The research findings can provide theoretical and technical references for remote sensing measurement of similar medicinal plant cultivation varieties.

## 1 Introduction

*Ligusticum chuanxiong* Hort. (LC) is one of the oldest and most popular herbal plants in the world, its dried rhizome has been widely used for centuries to promote blood circulation, regulate qi, dispel cold, and relieve pain (Wagner et al., 2011; Yuan et al., 2020). Since the Song Dynasty, the cultivation area of LC has been confined to Dujiangyan City in the Chengdu Plain of Sichuan Province (Kang et al., 2021). However, analogous to other medicinal plants of high economic value in China, the cultivation scale of LC has undergone rapid expansion in the past decade, leading to the emergence of new strip-shaped planting areas along the Minjiang River Basin (Fang et al., 2020). Farmers in Dujiangyan City usually manage an area of no more than 1 ha, but farmers in emerging planting areas, such as Mianzhu City, Meishan City, and Qionglai City, typically cultivate LC on areas spanning 10 to 100 ha (Peng et al., 2020). The significant expansion in planting scale presents considerable challenges for farmers to maintain detailed management. For example, if traditional manual field inspection is employed, where each inspector examines 1 ha per day, it would necessitate hiring 10 individuals to complete an inspection of 10 ha, resulting in high labor costs and the inability to ensure comprehensive coverage of the entire area. Moreover, farming system diversification, limited plot sizes, and crop variability contribute to the fragmentation of extensive LC cultivation areas, increasing the complexity for farmers to evaluate scattered LC fields on the ground. Therefore, the key challenge lies in how to efficiently aggregate data from extensive and scattered LC cultivation areas and utilize this data for growth status analysis, which is crucial for ensuring the high quality of medicinal materials.

The advancement of remote sensing technology has significantly transcended terrain limitations, offering hope for precise, repeatable, and swift identification of LC cultivation plots from an aerial perspective. Since the past decade, remote sensing technology has been widely used for extracting various crop cultivation plots, such as rice and corn (Guo and Ren, 2023; Li et al., 2024). Currently, satellite remote sensing technology is the mainstream method for studying crop spatial distribution (Khosravi, 2025; Rehman et al., 2025). Joel Segarra and colleagues utilized Sentinel-2 data to map wheat areas in the Burgos and Palencia regions of northern Spain (Segarra et al., 2023). Furthermore, Fan et al. explored the ability to classify crop types using Sentinel 2, Landsat 8, and GaoFen-1 data in a flat irrigation area in northwestern China, proving that classification accuracy will increase with the increase of input features (Fan et al., 2021). Satellite remote sensing provides advantages including broad spatial coverage and high information density, proving valuable for large-scale crop mapping in countries with dominant large-scale farming systems, flat terrains, and high mechanization levels such as the United States, Brazil, and Argentina (Fritz et al., 2019; Schwalbert et al., 2020). Conversely, this technology encounters limitations in southern China’s fragmented agricultural landscapes.

To effectively address the challenges of fragmented farmland monitoring, unmanned aerial vehicle remote sensing (UAVRS) technology demonstrates substantial potential. Current mainstream approaches primarily enhance classification accuracy through two pathways: sensor hardware upgrades and innovations in data processing algorithms. In the realm of sensors, hyperspectral imaging (HSI) and multispectral systems significantly improve spectral discrimination capabilities for ground objects by calculating various vegetation indices (e.g., NDVI, SAVI) (Wu et al., 2025; Zidi et al., 2025). At the algorithmic level, the development of object-based image analysis (OBIA) and deep learning semantic segmentation techniques has effectively overcome the salt-and-pepper noise problem inherent in traditional pixel-level classification (Yuan et al., 2021; Ez-zahouani et al., 2023). Research indicates that multidimensional feature fusion constitutes a core strategy for accuracy enhancement: The HSI-Trans U-Net model developed by Niu et al. (2022), which fuses spatial-spectral features from hyperspectral images, achieved a crop classification accuracy of 86.05%. Wei et al. (2019) integrated spectral information, spatial context, and positional features within a conditional random field framework, elevating accuracy to 98.07%. Xu et al. (2023)further validated the efficacy of fusing spectral-textural features in time-series imagery for overcoming the limitations of single-date classification. Notably, low-cost RGB sensors also exhibit significant potential through innovative feature engineering (Pádua et al., 2022; Han et al., 2024). Wang et al. (2025) demonstrated that solely utilizing UAV-based RGB imagery and fusing color features, textural features, and lightweight convolutional features, achieved a 99% classification accuracy for rice-wheat rotation progress. This performance substantially surpassed the accuracy of single-color-feature models (85.3%), with single-image processing requiring only 1.25 seconds. This reveals that optimized feature combination strategies using RGB sensors can rival or even outperform expensive hyperspectral systems. However, it should be noted that existing high-accuracy methods still possess significant limitations. These include high equipment dependency (hyperspectral/multispectral sensors costing 5 to 10 times more than conventional RGB cameras (Tian et al., 2022), considerable operational complexity (multi-temporal data acquisition requiring precise phenological synchronization (Zhou et al., 2023), and a narrow focus on specific research subjects. Studies predominantly concentrate on major staple crops such as rice, maize, and wheat, while research on medicinal plant parcel identification remains scarce. Particularly, the identification of LC, an important characteristic medicinal plant in Southwest China, has yet to be reported. Given the absence of prior dedicated studies on LC plots identification and building upon the established principle that enhanced input features improve classification accuracy, this study pioneers the extraction of LC planting plots using exclusively low-cost UAV-based RGB imagery. Our primary objective is to determine the optimal feature combination and classification methodology through rigorous evaluation of accuracy metrics. The findings are expected to serve as a reference for identifying medicinal plant cultivation plots utilizing UAV-based RGB imagery. The research goals consist of three parts: (1) comparing the extraction outcomes of LC planting areas based on various image feature variable combinations and selecting the most precise one; (2) evaluating the extraction effectiveness using pixel classification algorithms and object-oriented classification algorithms and selecting the most accurate classification algorithm; and (3) employing data from other LC regions and examining the extraction results of LC planting areas using UAV-based RGB imagery and combined feature variables to validate the applicability of the proposed extraction method in this study.2. Study area and data source.

## 2 Study area and data source

### 2.1 Study area

Site 1 (S1) is located in Dujiangyan City, Chengdu, Sichuan Province, China. The site lies in the central part of Sichuan Province, at the intersection of the upper and middle reaches of the Minjiang River, between 103°37′–103°42′E and 30°46′–30°52′ N. This area belongs to the subtropical humid monsoon climate zone of the Sichuan Basin and features low temperatures, ample rainfall, and limited sunlight. The area is particularly suited for the growth of LC, which has been a genuine producing area since the Song Dynasty and produces the optimal quality of LC. The research area covers 142,157.86 m^2^, with a relatively concentrated cultivation area of LC measuring 30,161.97 m^2^.

**Figure 1:**
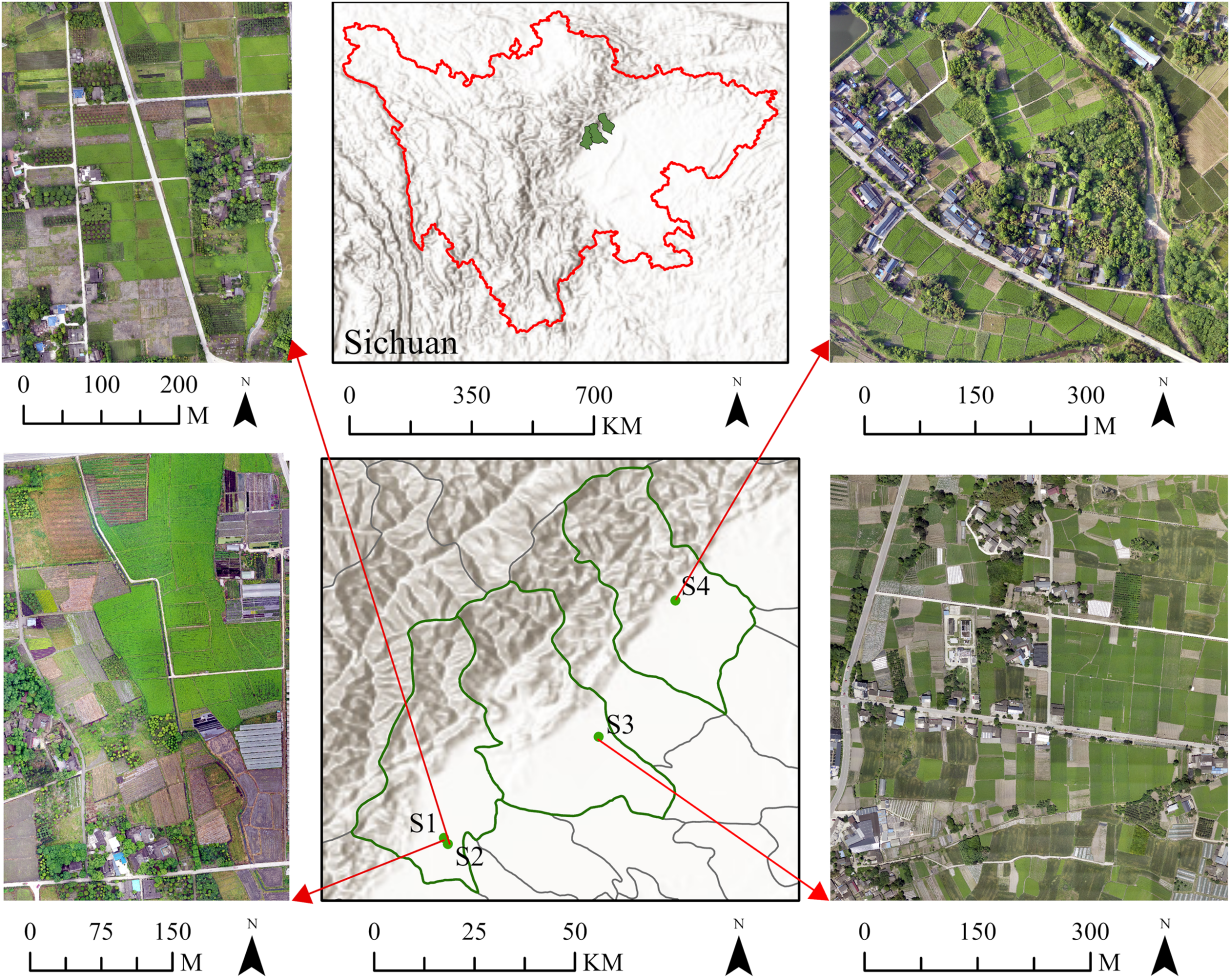
Geographic location of the study area.

To investigate the adaptability of various methods, three additional LC cultivation areas were selected as verification sites: Site 2 (S2) is located in Dujiangyan City with an area size of 176,508.36 m^2^ and a cultivation area of LC measuring 31,841.85 m^2^. The cultivation plots here are relatively concentrated, with minimal occurrences of intermingling with forest land. Site 3 (S3) is found in Pengzhou City, with an area size of 298,678.20 m^2^ and a cultivation area of LC measuring 81,284.51 m^2^. The plots are well defined and exhibit few instances of blending with forest land. Site 4 (S4) is situated in Mianzhu City with an area size of 263,629.88 m^2^, within which the cultivation area of LC measures 46,252.38 m^2^. The plots are irregularly shaped and experience greater encroachment by forest land. The geographical location of the four study areas in this research is depicted in **Figure 1**.

### 2.2 UAV image acquisition and preprocessing

The aerial platform utilized in this study was the DJI Mavic 2 Pro, which is battery-powered and has a take-off weight of 907 g. The endurance of the UAV lasts for approximately 30 min. The camera model employed is the Hasselblad L1D-20c, boasting a pixel count of 20 million. At an altitude of 100 m, the ground resolution reaches 2.38 cm/pixel, effectively meeting the demands of this project for capturing RGB images. DJI GS PRO was applied to plan the aerial survey and create a flight itinerary based on the actual conditions of the site. Flying at an altitude of 100 m and a speed of 8.6 m/s, along with headings and lateral overlap percentages of 75% and 65%, respectively, ensured efficient coverage of the surveyed area. Data collection occurred on April 25, between 11:48 am and 12:22 pm, and again on April 27, from 13:18 pm to 13:34 pm and from 17:00 pm to 17:22 pm, as S1, S2, S3, and S4 datasets, respectively.

To process the collected UAV images, Agisoft Metashape Pro (Agisoft LLC, St. Petersburg, Russia) was employed to implement the Structure from Motion workflow. After cropping out the parts with poor edge quality in the software, the coordinate system was converted to World Geodetic System 84-Universal Transverse Mercator coordinate system Zone 48N (WGS 84/UTM zone 48N), and the spatial resolution was resampled to 0.10 m/pixel. Digital orthophoto map and digital elevation model (DEM) images were output separately.

### 2.3 Reference data

In the course of conducting a survey of land cover types in the study area, concurrent UAV image acquisition was employed. This methodology involved combining field observations with UAV imagery to visually interpret various land cover types and establish sampling datasets based on these classifications. Through field investigations, the primary land cover types detected in each research area were categorized as follows: LC, forest, other plants, bare land, and construction land. Consequently, this study meticulously considered the spectral characteristics of UAV images and field investigation findings and bifurcated the land cover types within the study area into five categories. To facilitate analysis, we processed drone imagery from four study areas (S1–S4) using ENVI 5.3 and ArcGIS Pro 10.1 to generate samples representing five distinct land cover types for classification model training and validation through the following workflow: first, distribution polygons for each land cover class were delineated across S1–S4 imagery using ENVI 5.3’s Region of Interest (ROI) Tool and exported as Shapefiles; subsequently, these vectors were imported into ArcGIS Pro 10.1 where a random point generation tool applied a spatial separation criterion (>3 meters with 0.2m point radius) to create initial sample points; finally, manual refinement eliminated incomplete boundary points and low-quality samples to derive valid sample points, with per-area totals (S1: 3200, S2: 3500, S3: 4540, S4: 2260) summarized in **Table 1**, after which these points were partitioned into training/validation sets implementing 5-fold cross-validation during model training to enable comprehensive evaluation.

**Table 1:** The number of training and validation samples in this study.

Sample types	LC	Forest	Other Plants	Bare land	Construction land
Training (S1)	100	100	100	100	100
Validation (S1)	600	600	600	450	450
Total (S1)	700	700	700	550	550
Training (S2)	140	120	100	40	50
Validation (S2)	900	500	750	450	450
Total (S2)	1040	620	850	490	500
Training (S3)	150	30	30	150	130
Validation (S3)	1050	600	900	750	750
Total (S3)	1200	630	930	900	880
Training(S4)	160	150	130	60	80
Validation(S4)	450	450	360	180	240
Total(S4)	610	600	490	240	320

## 3 Methods

**Figure 2:**
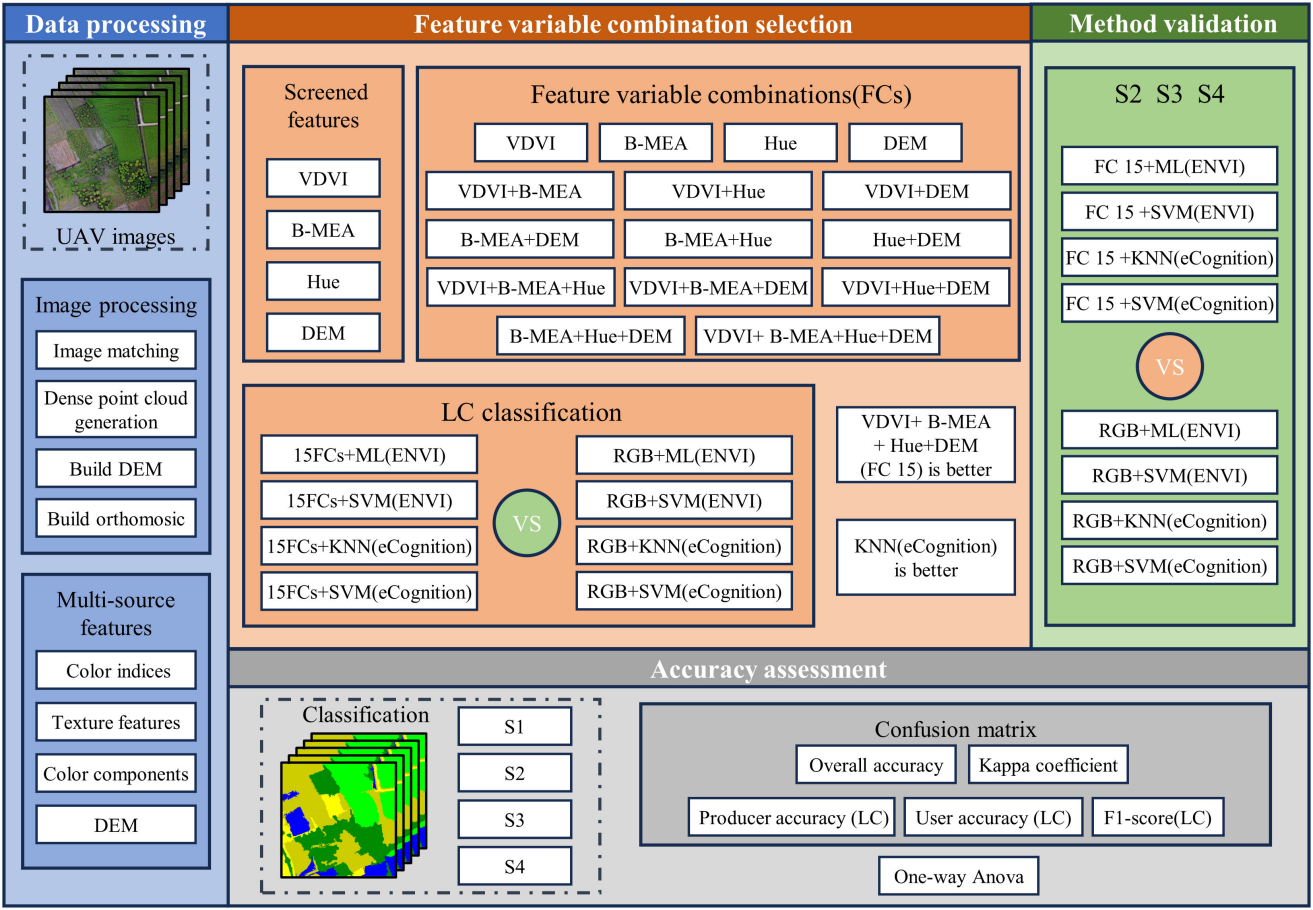
Workflow of this study: VDVI, B-MEA, Hue, and DEM denote the Visible-band Difference Vegetation Index, the Mean texture feature extracted from the blue channel of RGB imagery, the Hue component in the HSV or HLS color space, and the Digital Elevation Model, respectively; while ML, KNN, and SVM represent the machine learning classifiers: Maximum Likelihood, k-nearest neighbor, and Support Vector Machine.

This study evaluated the effectiveness of various combinations of feature variables in conjunction with pixel-based and object-oriented classification models for the classification of LC cultivation plots. The main steps include the following: (1) constructing multisource features based on UAV images. These features were obtained from color indices, texture features, and color components extracted from the images. (2) Selecting representative image feature variables from color indices, texture features, and color components and constructing feature variable combinations. These variable combinations were then classified using pixel-based and object-oriented machine learning algorithms, and their performance was compared against those obtained using RGB images alone. This step aimed to identify the most effective feature variable combinations for classification. (3) Applying the optimal feature variable combination method identified in step (2) to RGB images acquired from S2, S3, and S4. The resulting classifications were then compared against those obtained using RGB images to assess the stability of the proposed approach across different scenes and conditions. The workflow of this study is depicted in **Figure 2**.

### 3.1 Image feature variable screening

#### 3.1.1 Image feature variable construction

To enhance the separability of different objects by amplifying the spectral information difference of different objects, a series of color indices were constructed based on RGB remote sensing images (Woebbecke et al., 1995). Although there are few studies on color index construction based on RGB images, 12 color indices were selected for this study after reviewing relevant literature in recent years (**Table 2**).

**Table 2:** Color indices selected in this study.

Color Index	Formula	Reference
green chromatic coordinate (GCC)	G/(R+G+B)	Richardson et al. (2007)
red chromatic coordinate (RCC)	R/(R+G+B)	Richardson et al. (2009)
blue chromatic coordinate (BCC)	B/(R+G+B)	Richardson et al. (2007)
green–red vegetation index (GRVI)	(G−R)/(G+R)	Kawashima and Nakatani (1998)
woebbecke index (WI)	(G−B)/(R−G)	Woebbecke et al. (1995)
green leaf index (GLI)	(2×G−R−B)/(2×G+R+B)	Booth et al. (2006)
visible atmospherically resistance index (VARI)	(G−R)/(G+R−B)	Gitelson et al. (2002)
excess red vegetation index (EXR)	1.4×RCC−GCC	Meyer and Neto (2008)
excess green vegetation index (EXG)	2×GCC−RCC−BCC	Guijarro et al. (2011)
excess blue vegetation index (EXB)	1.4×BCC−GCC	Guijarro et al. (2011)
color index of vegetation (CIVE)	0.441×R−0.881×G+0.385×B+18.78745	Kataoka et al. (2003)
visible-band difference vegetation index (VDVI)	(G-B-R)/(G+B+R)	Wang et al. (2015)

Texture features are one of the most commonly used features for image classification because different ground objects have different texture information (Ma and Manjunath, 1996). This study selects the Gray-level Co-occurrence Matrix (GLCM) (Soh and Tsatsoulis, 1999) as a widely used texture feature to calculate the spatial correlation of image gray sets. GLCM includes eight kinds of features: Mean (MEA), Variance (VAR), Homogeneity (HOM), Contrast (CON), Dissimilarity (DIS), Entropy (ENT), Second Moment (SEM), and Correlation (COR) (Equations 1–8). A total of 24 GLCM features were extracted based on 3 bands (R, G, and B) of RGB images, of which the parameters were used in the default setting, such as the window size of 3 × 3, the angle of 0°, and the quantization level of 64. GLCM textural features obtained for this study are as follows:

(1) $$Mean=\sum_{i=1}^{N_{g}}\sum_{j=1}^{N_{g}}i*P\;\left(i,j\right)$$

(2) $$Variance=\sum_{i=1}^{N_{g}}\sum_{j=1}^{N_{g}}\;{\left(i-\mu \right)}^{2}P\;\left(i,j\right)$$

(3) $$Homogeneity=\sum_{i=1}^{N_{g}}\sum_{j=1}^{N_{g}}\frac{1}{1+\;{\left(i-j\right)}^{2}}P\;\left(i,j\right)$$

(4) $$Contrast=\sum_{i=1}^{N_{g}}\sum_{j=1}^{N_{g}}\;{\left(i-j\right)}^{2}P\;\left(i,j\right)$$

(5) $$Dissimilarity=\sum_{i=1}^{N_{g}}\sum_{j=1}^{N_{g}}\left|i-j\right|P\;\left(i,j\right)$$

(6) $$Entropy=\sum_{i=1}^{N_{g}}\sum_{j=1}^{N_{g}}P\;\left(i,j\right)\log\;\left(P\;\left(i,j\right)\right)$$

(7) $$Second\;Moment=\sum_{i=1}^{N_{g}}\sum_{j=1}^{N_{g}}{\left\{P\;\left(i,j\right)\right\}}^{2}$$

(8) $$Correlation=\frac{\sum_{i}\sum_{j}\;\left(i,j\right)P\;\left(i,j\right)-\mu_{x}\mu_{y}}{\sigma_{x}\sigma_{y}}$$

During aerial photography of UAVs in outdoor fields, changes in light intensity are inevitable and different for different types of objects. The HSV and HLS color space models can offset the color differences of the same object caused by differences in light intensity by independently adjusting brightness, chroma, and color (Demarty and Beucher, 1998; Sural et al., 2002). This makes them useful for vegetation type classification and extraction. The HLS color space simulates colors by combining the three color channels of Hue, Lightness (LIT), and Saturation (SAT), while the HSV color space simulates colors by combining the three color channels of Hue, Saturation, and Value (VAL). After converting the RGB image to obtain two types of color spaces, the four obtained color components are used as image feature variables to participate in the image feature variable screening process.

Moreover, taking into account that the variation in vegetation height between LC and forest land, as well as other vegetative types, might contribute to classification, the DEM generated during the orthogonalization process was employed to combine feature variables with additional variables.

#### 3.1.2 Image feature variable selection

**Figure 3:**
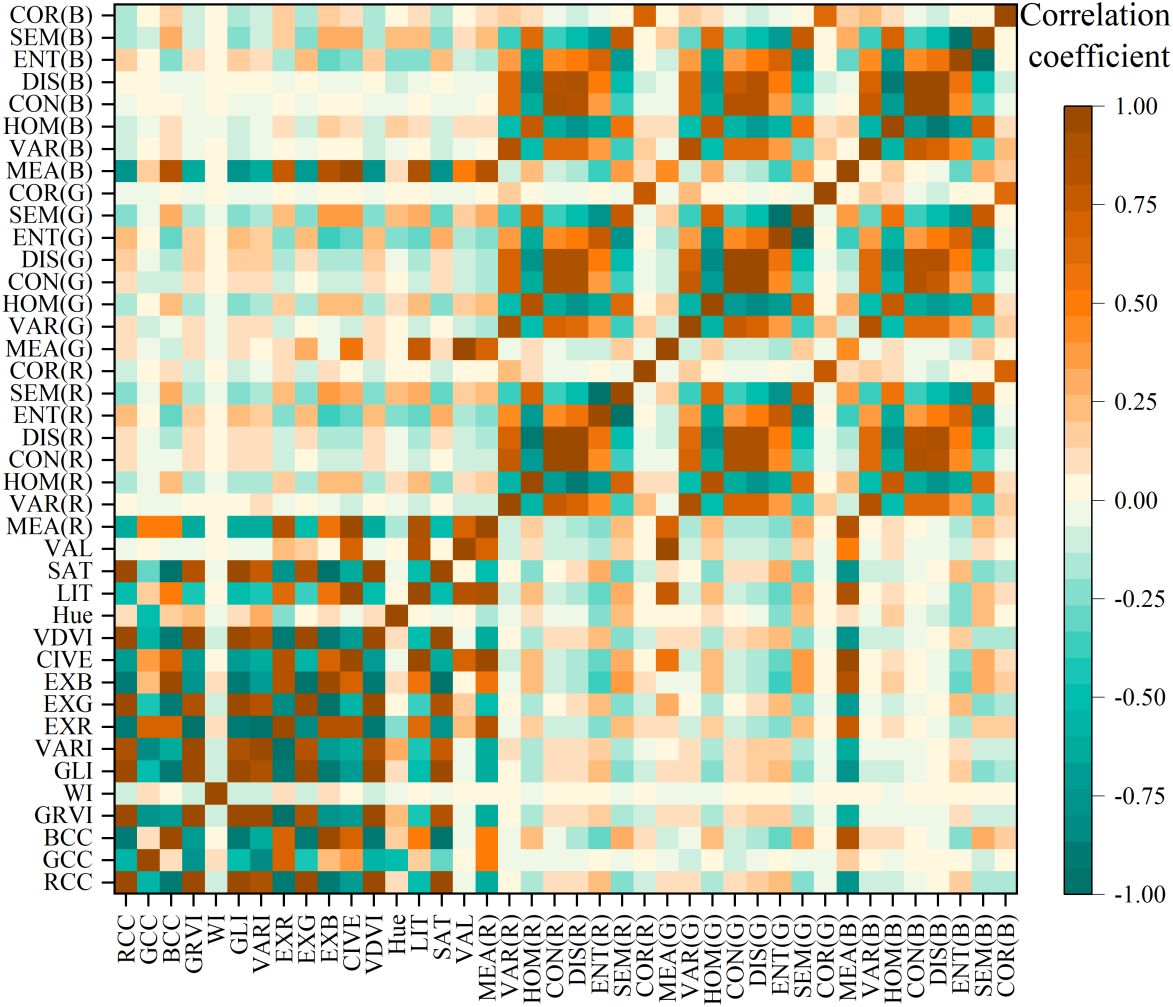
Heatmap for the Pearson’s correlation analysis of multi-source features.

If there is a high correlation between various features in the multidimensional image, it can lead to poor classification results. Additionally, having an excessive number of features involved in the classification process can greatly increase the demand for computing resources, thereby reducing the classification efficiency. To mitigate this challenge, we performed a Pearson correlation coefficient analysis on the 12 calculated color indices, 24 texture features, and 4 color components, as shown in **Figure 3**. The analysis revealed a high correlation between different color indices, which may be because they are all used to represent the degree of reflectivity of the surface of the object. Similarly, different texture features and color components show high correlation, suggesting that there is redundant information among similar variables. Consequently, we suggest selecting only one feature variable from each of the three categories of feature variables for integration to enhance classification precision while minimizing the time needed for classification.

To assess the contribution of the 12 computed color indices, 24 texture features, and 4 color components in promoting the separability of different land types and LC fields and to select the most suitable feature variables for classification, we statistically analyzed the average values of different image feature variables in each land type based on sample data. Subsequently, we calculated the interclass difference scores $D_{w}$ between samples of each type of land and samples of LC fields using the following formula (Equation 9):

(9) $$D_{w}=ABS\left(\frac{M_{c}-M_{n}}{M_{c}}\right)*100\%$$

where $D_{w}$ is the interclass difference score of the spectral mean of other land samples and LC samples, Mc is the spectral mean of LC samples, and Mn is the spectral mean of other land samples.

To assess the effectiveness of various combinations of image features in classifying LC cultivation plots and identify the optimal combination of variable features, this study implemented four classification scenarios, as follows: (1) Scenario 1: Utilizing only one of the four category feature variables; (2) Scenario 2: Combining two feature variables; (3) Scenario 3: Combining three feature variables; (4) Scenario 4: Integrating all four feature variables (CI, CC, TF, and DEM). The specific groupings are presented in **Table 3**.

**Table 3:** Feature combinations designed in this study.

Name	Feature combination	Feature Contented
Feature combination 1 (FC 1)	CI	1
Feature combination 2 (FC 2)	CS	1
Feature combination 3 (FC 3)	GLCM	1
Feature combination 4 (FC 4)	DEM	1
Feature combination 5 (FC 5)	CI+CS	2
Feature combination 6 (FC 6)	CI+GLCM	2
Feature combination 7 (FC 7)	CI+DEM	2
Feature combination 8 (FC 8)	CS+GLCM	2
Feature combination 9 (FC 9)	CS+DEM	2
Feature combination 10 (FC 10)	GLCM+DEM	2
Feature combination 11 (FC 11)	CI+CS+GLCM	3
Feature combination 12 (FC 12)	CI+CS+DEM	3
Feature combination 13 (FC 13)	CI+GLCM+DEM	3
Feature combination 14 (FC 14)	CS+GLCM+DEM	3
Feature combination 15 (FC 15)	CI+CS+GLCM+DEM	4

### 3.2 Feature variable combination selection

To assess the viability of incorporating various feature variables into enhancing the accuracy of LC classification, we compared the performance of three widely adopted machine learning classifiers: maximum likelihood (ML) (Lillesand et al., 2015), k-nearest neighbor (KNN) (eCognition, 2013), and support vector machine (SVM) (Vapnik, 1999). These classifiers are typically utilized in the literature for remote sensing image classification. By evaluating the performance of these classifiers on differently combined feature variable images, we measured the performance of different image-variable feature combinations in improving classification accuracy. The strengths of KNN and ML consist of their simplicity and capacity to execute high classification efficiency on low-performance computing devices. Conversely, SVM demonstrates outstanding performance in high-dimensional spaces, making it an appropriate choice for classification tasks in intricate scenarios. Therefore, we selected KNN and SVM for object-oriented classification tasks, whereas ML and SVM served as classification models for pixel-based classification tasks. All classifiers adhered to their default specifications throughout usage, and all models underwent 5-fold cross-validation during the training process to evaluate their generalization abilities.

#### 3.2.1 Pixel-based classification

Pixel-based classification is a fundamental concept in traditional remote sensing (Whiteside et al., 2011). After identifying the types of land objects in the research area via the interpretation of the research area image and field survey data, representative pixels of each type of land object were selected as training samples, and the ML classifier and SVM classifier were used for land object classification, respectively. Since the classification results obtained from supervised classification are preliminary results, frequently featuring small patches, it is crucial to refine these results from a practical application viewpoint. Thus, we utilize the majority/minority analysis tool to eliminate small patches, obtain post classification processing results, and export the classification outcomes in TIF format. Finally, we calculate the proportion and area attributes of each type of land object within each classification result. The pixel-based classification tasks conducted in this study were executed using ENVI 5.6.3.

#### 3.2.2 Object-oriented classification

Object-oriented classification is a method of image classification that differs from traditional pixel-based classification (Yan et al., 2006). This method segments the image into coherent pixel groups characterized by similar features such as spectrum, texture, and shape, i.e., image objects. Subsequently, the method performs supervised classification based on the spectral, texture, shape, and other feature differences of different land-type objects. Since it classifies block-like pixel sets, object-oriented classification offers significant computational efficiency improvements while effectively avoiding salt and pepper artifacts (Bhaskaran et al., 2010; Tassi and Vizzari, 2020). The implementation of object-oriented classification mainly includes two core steps: multiscale segmentation and classification. Optimal segmentation outcomes prevent the formation of objects containing solely a single land type, minimizing fragmentation and more accurately reflecting the shape features of individual lands, such as farmland areas.

**Figure 4:**
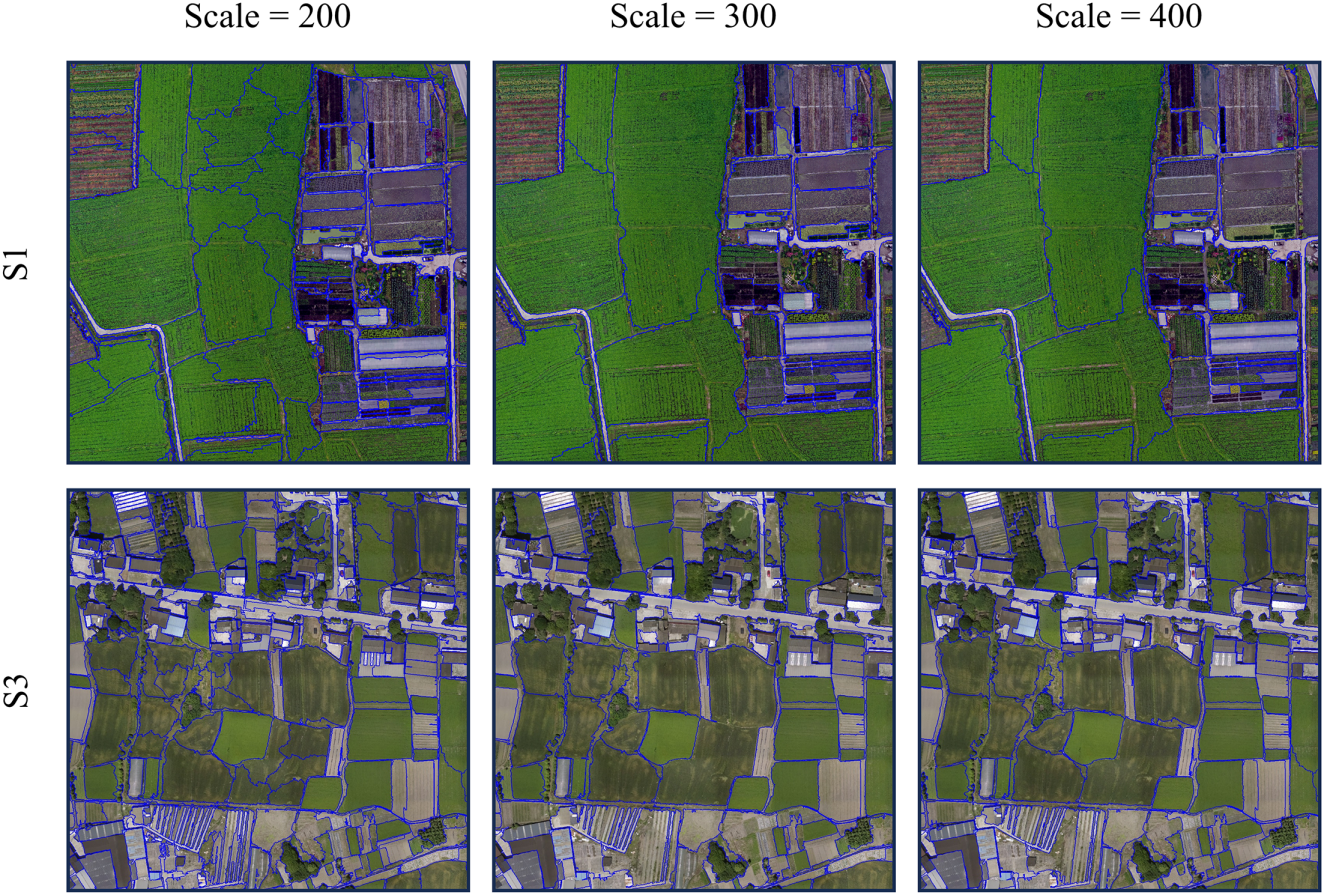
Visual comparison of multiscale segmentation results. The blue lines are the boundaries of multiscale segmentation.

The crucial parameters of the multiscale segmentation algorithm include segmentation scale, shape factor, compactness factor, and image layer weight. Given the flat terrain and interconnected crops within the research area, the shape factor was set to 0.3, the compactness factor was set to 0.5, and all image layer weights were set to 1. Based on optimizing these initial three parameters, we employed the ESP2 evaluation tool (Drăguţ et al., 2014) to determine the optimal segmentation scale based on the RGB images of S1 with a smaller coverage area and S3 with a broader scope. The optimal segmentation scale was determined to be 300, and the results were compared with those obtained using segmentation scales of 200 and 400. The outcomes indicated that when the segmentation scale is set to 300, the pixels affiliated with the same land object category via multiscale segmentation exhibit spectrum similarities in spectrum, texture, shape, and space, whereas dissimilar land objects demonstrate heterogeneity (**Figure 4**). Finally, each feature combination image is classified separately using the KNN classifier and SVM classifier. The boundaries of contiguous, same-type land in the classification results are eliminated and outputted in TIF format. The object-oriented classification endeavors undertaken in this study were executed utilizing eCognition 9.0.

### 3.3 Method validation

To assess whether the feature variable combination obtained can maintain the accuracy advantage in LC cultivation plot extraction across different regions, we developed the most effective feature variable combination based on RGB images from S2, S3, and S4. Subsequently, we implemented pixel-based classification and object-oriented classification algorithms to classify land use and compared their outcomes with those obtained using RGB images to validate the potential of transferring the optimal feature variable combination to other areas.

### 3.4 Evaluation indicators

To assess the performance of the land-use classification results, we employed the confusion matrix (Congalton and Green, 2019) to compare our classifications with the corresponding verification samples of land-use categories. Based on the computed confusion matrix, overall accuracy and Kappa coefficient were used to evaluate the overall accuracy of the classification, while LC’s producer accuracy, user accuracy, and F1 score were calculated to evaluate the classification accuracy of LC (Equations 10–14). To objectively compare the performance of different feature combinations and identify the optimal solution, we established a composite scoring system with equal weighting: 0.2×OA + 0.2×Kappa + 0.2×PA (LC) + 0.2×UA (LC) + 0.2×F1 (LC), ensuring balanced consideration of all metrics. Furthermore, one-way analysis of variance (ANOVA) was implemented to determine whether there are any significant differences in classification accuracy among various feature combinations. Subsequently, Tukey’s Honestly Significant Difference (HSD) test was applied to perform pairwise comparisons between the composite scores of each feature combination and the baseline RGB feature combination. The analysis results are presented in a ranking table sorted in descending order of composite score. Feature combinations demonstrating statistically significant superiority over the RGB baseline (p< 0.05) are marked with an asterisk (*). Complete ranking results are provided in **Supplementary Tables S1-S4**.

(10) $$overall\;accuracy=\frac{1}{n}\sum_{i=1}^{r}x_{ij\;}\;$$

(11) $$kappa\;coefficient=\frac{n*\sum_{i=1}^{r}x_{ij}-\sum_{i=1}^{r}\;\left(x_{i}*x_{j}\right)}{n^{2}-\sum_{i=1}^{r}\;\left(x_{i}*x_{j}\right)}$$

(12) $$producer\;accuracy=\frac{x_{ij}}{x_{j}}*100\%$$

(13) $$user\;accuracy=\frac{x_{ij}}{x_{i}}*100\%$$

(14) $$F1\;score=\frac{2*PA_{i}*UA_{i}}{PA_{i}+UA_{i}}*100\%$$

Where n is the total number of samples, r is the number of confusion matrix rows and columns, $x_{ij}$ is the value of row i and column j in the confusion matrix, $x_{i}$ is the sum of row i in the confusion matrix, $x_{j}\;$ is the sum of column j in the confusion matrix.

## 4 Results

### 4.1 Image feature variable selection

**Table 4** shows the interclass difference scores $D_{w}$ of the spectral mean of each land class sample compared with LC across the 12 color index features. In the GCC, RCC, GLI, EXG, and CIVE images, the difference scores between each type of land use and LC were relatively small, all less than 100%, with some instances where the pixel mean of construction land was comparable with other plants, which was not conducive to land classification. In the RCC, GRVI, WI, VARI, EXR, EXB, and VDVI images, the means of the WI feature were similar for all three classes: other plants, bare land, and construction land; the BCC, EXG, and EXB features exhibited similar means for forest and other plants; the GRVI feature had similar means for other plants and bare land; and the VARI feature displayed similar means for construction land and forests. These similarities did not contribute positively to land classification accuracy. By analyzing the mean differences between each type of land-use sample in the EXR and VDVI images, we found that the difference between construction land and other plants in the EXR image was not significant, whereas the separation among various types of land-use in the VDVI image was higher. Consequently, VDVI was selected as the feature color index to participate in the study of feature combinations for land classification.

**Table 4:** Inter-class difference scores in different color index features of LC and other land-use types.

Feature	Forest	Other Plants	Bare land	Construction land
GCC	11.11%	22.22%	35.19%	37.04%
RCC	6.25%	15.63%	12.50%	3.13%
BCC	53.33%	40.00%	93.33%	133.33%
GRVI	11.54%	73.08%	103.85%	80.77%
WI	9.60%	122.03%	112.99%	114.12%
GLI	27.50%	52.50%	90.00%	97.50%
VARI	6.45%	67.74%	103.23%	0.00%
EXR	52.00%	209.76%	300.04%	238.32%
EXG	35.03%	52.59%	88.58%	96.04%
EXB	54.92%	58.75%	121.68%	171.65%
CIVE	5.55%	27.74%	55.59%	78.07%
VDVI	162.50%	287.50%	455.00%	500.00%

In **Table 5**, the interclass difference scores $\;D_{w}$ of each land class sample and LC were presented for the 24 texture feature variables. Notably, in the R-MEA, R-ENT, R-SEM, G-MEA, G-ENT, G-SEM, B-HOM, B-CON, B-DIS, B-ENT, and B-SEM images, there were instances where the interclass difference scores between land-use types and LC were less than 10%, rendering them unsuitable for land classification. Additionally, in the R-VAR, R-HOM, R-CON, R-DIS, R-COR, G-HOM, G-DIS, G-COR, B-VAR, and B-COR images, the means of other plant samples were found to be closely aligned with those of forest samples, whereas the means of construction land samples were comparable with those of other plant samples. These situations were unhelpful for effective land classification. Conversely, only in the G-VAR, G-CON, and B-MEA images did the means of each type of land-use sample exhibit distinguishable differences. Combining the interclass difference scores between the means of each type of land-use sample in each feature image and the mean of LC samples, B-MEA was selected as the texture feature for feature combination research in land-use classification.

**Table 5:** Inter-class difference scores in different texture features of LC and other land-use types.

Feature	Forest	Other Plants	Bare land	Construction land
R-MEA	8.50%	28.77%	44.14%	43.28%
R-VAR	93.06%	90.97%	13.54%	34.38%
R-HOM	10.53%	13.16%	18.42%	44.74%
R-CON	75.99%	77.63%	22.53%	36.02%
R-DIS	27.60%	30.73%	15.63%	33.33%
R-ENT	1.48%	1.97%	4.93%	18.23%
R-SEM	0.00%	7.14%	14.29%	64.29%
R-COR	93.75%	81.25%	100.00%	87.50%
G-MEA	16.18%	13.60%	16.97%	6.77%
G-VAR	142.28%	104.41%	13.24%	33.82%
G-HOM	20.00%	17.50%	15.00%	45.00%
G-CON	120.18%	93.81%	20.53%	34.34%
G-DIS	44.02%	37.50%	14.67%	34.24%
G-ENT	2.97%	2.97%	4.95%	22.28%
G-SEM	7.14%	7.14%	14.29%	85.71%
G-COR	100.00%	55.56%	66.67%	44.44%
B-MEA	48.21%	82.53%	157.16%	267.68%
B-VAR	106.67%	116.67%	21.90%	15.71%
B-HOM	16.28%	20.93%	4.65%	34.88%
B-CON	89.62%	101.32%	6.40%	19.87%
B-DIS	34.55%	40.61%	0.61%	26.67%
B-ENT	3.03%	4.55%	2.53%	19.70%
B-SEM	6.67%	13.33%	6.67%	66.67%
B-COR	160.00%	140.00%	200.00%	160.00%

**Table 6** shows the interclass difference scores $D_{w}$ between various land-use types and LC in the four color component feature variables. In the Light and Value images, some instances exhibited minimal differences between land-use classes, making it challenging for accurate land-use classification. Additionally, while the sample mean of bare land in the Saturation image closely resembled that of construction land and the mean of forest lands was similar to that of other plants, this lack of differentiation was unfavorable for land classification purposes. Conversely, only in the Hue image does each land-use type exhibit distinct sample means; hence, Hue was selected as the color space feature for feature combination research in land-use classification.

**Table 6:** Inter-class difference scores in different color space features of LC and other land-use types.

Feature	Forest	Other Plants	Bare land	Construction land
Hue	9.27%	19.78%	37.46%	73.87%
Light	2.78%	2.78%	22.22%	41.67%
Saturation	36.84%	40.35%	78.95%	78.95%
Value	16.07%	12.50%	12.50%	1.79%

### 4.2 Classification results of feature variable combinations

**Figure 5:**
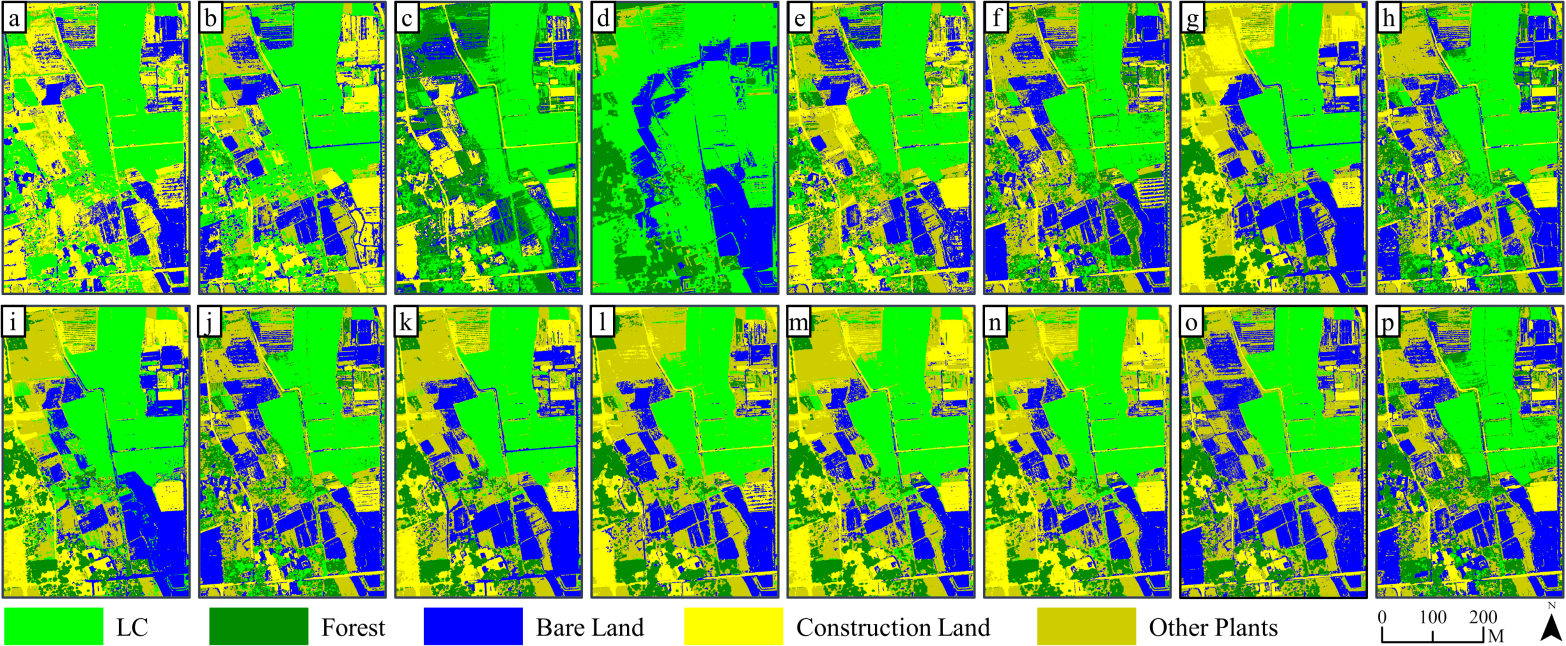
Results of ML (pixel-based) classification on 15 kinds of feature combinations: **(a–o)** are classification results based on FC1~FC 15, while **(p)** is the classification results based on RGB image.

**Figure 6:**
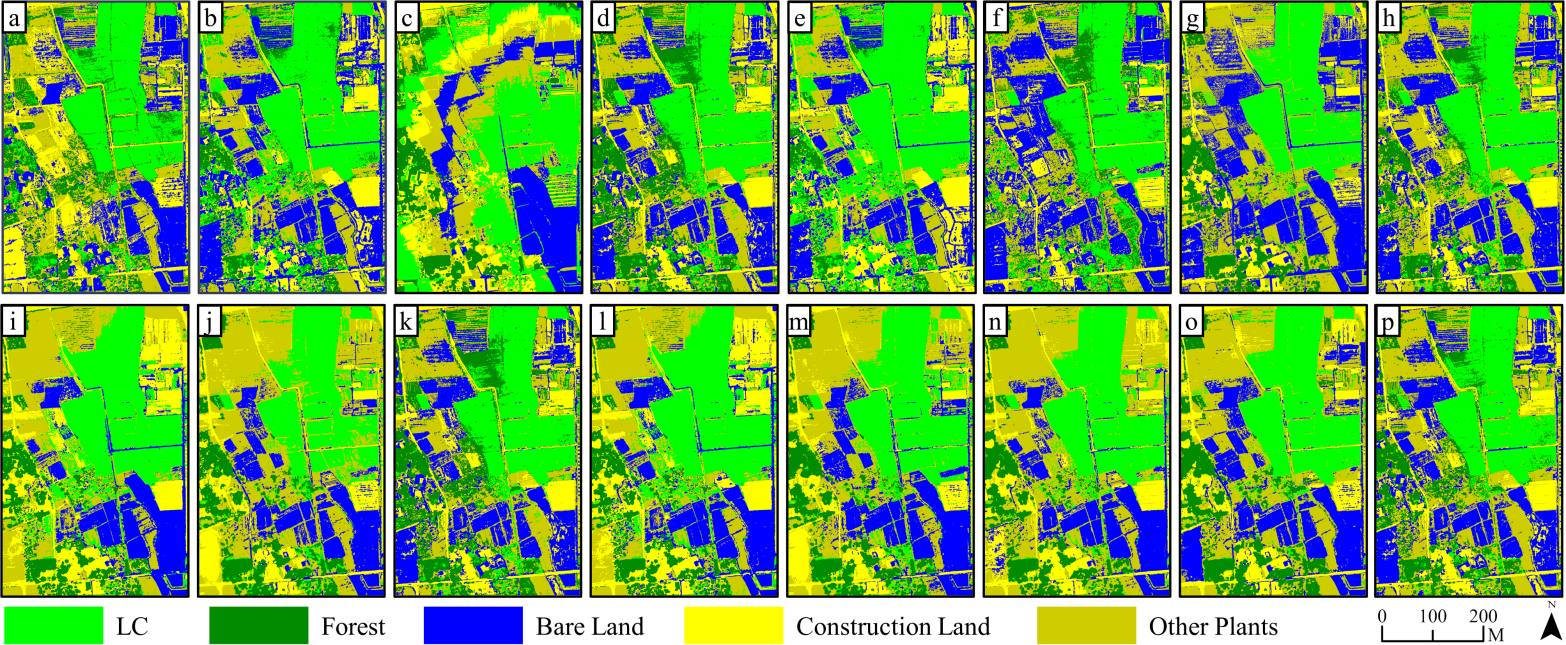
Results of SVM (pixel-based) classification on 15 kinds of feature combinations: **(a–o)** are classification results based on FC1~FC 15, while **(p)** is the classification results based on RGB image.

**Figures 5** and **6** show that as the number and diversity of features participating increased, the classification accuracy of land objects and the extraction accuracy of the LC cultivation area demonstrated a consistent improvement under both pixel-based classification methods.

Specifically, it showed that it is difficult to effectively distinguish land objects based solely on one of the four feature variables. For example, since FC 4 only contained elevation information, classification errors for other land objects were apparent except for forest recognition, which performed well, and a large area of pixels being misclassified as LC. Integrating two features for classification notably enhanced the classification outcomes when compared with using only one variable, with the exception of FC 9 and 10. Furthermore, the combination of three features led to even better classification results for FC 11 through 14, with FC 13 demonstrating the greatest reduction in classification errors and salt and pepper effects within the extracted LC cultivation area. Finally, incorporating all four features into FC 15 produced satisfactory outcomes in land classification and LC cultivation area extraction.

**Figure 7:**
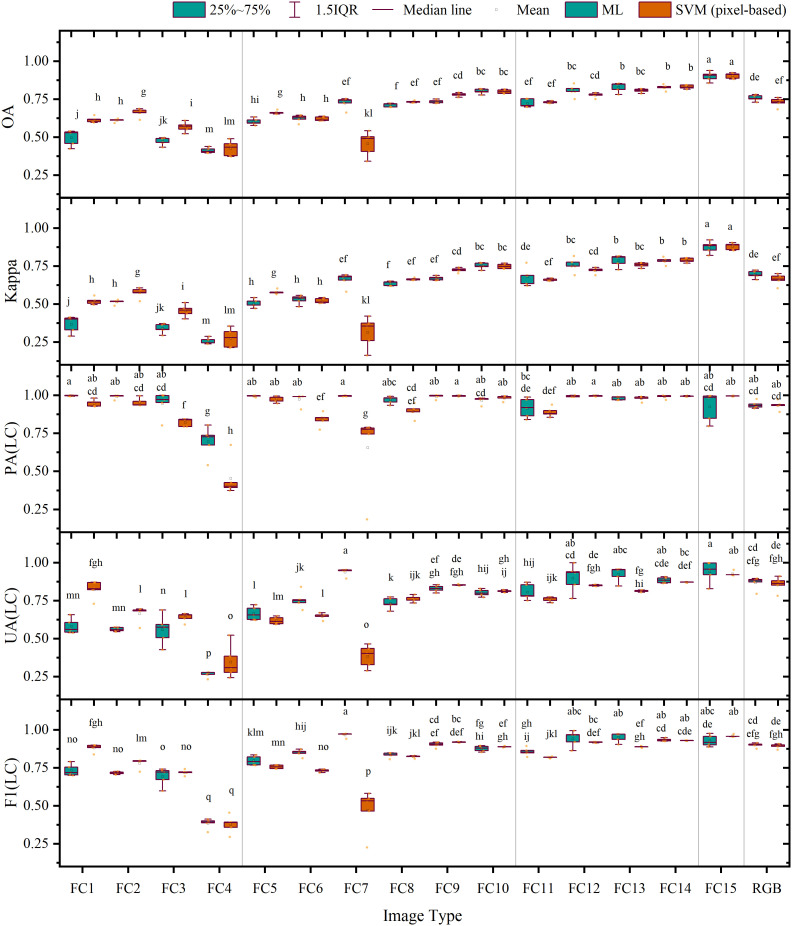
Accuracy evaluation of the results of pixel-based classification: the letters on the top of charts shows that the differences are significant.

Furthermore, **Figure 7** shows that the average overall accuracies of FC 1–4 classified using a single feature were all below 70.00%, and their average Kappa coefficients were all lower than 0.60. Among these, the accuracies and Kappa coefficients of feature combination 4 based on the DEM were the lowest. For FC 5–10 combined using two features, except for FC 7 classified using SVM, the average overall accuracies ranged from 60.00% to 80.00%, and their average Kappa coefficients lay between 0.50 and 0.75, showing limited improvement over the classification accuracy of a single feature. For FC 11–14 combined using three types of features, except for feature combination 11, the average overall accuracies were all above 80.00%, and their average Kappa coefficients exceeded 0.80. Among them, the highest average overall accuracies and Kappa coefficient values were achieved by feature combination 14 with average overall accuracies of 82.70% (ML) and 83.07% (SVM) and average Kappa coefficients of 0.78 (ML) and 0.79 (SVM), respectively, second only to FC 15 involving four features. The composite scoring system ranked FC15 as the optimal combination in both classifiers (ML composite score: 0.91; SVM composite score: 0.93; see **Supplementary Table S1, S2** for composite scores and full rankings of all feature combinations). The average overall accuracy and Kappa coefficient of FC 15 reached 90.00% (ML), 90.43% (SVM), 0.87 (ML), and 0.88 (SVM), respectively, demonstrating a significant improvement of 14.26% (ML), 17.44% (SVM), 0.18 (ML), and 0.22 (SVM) compared with the results based on RGB image accuracy. These findings suggest that the increase in the number and types of features involved can significantly enhance land classification accuracy.

In assessing LC cultivation plot extraction using the ML classifier, FC 4 (DEM) achieved the lowest producer accuracy (69.67%). In contrast, all other features yielded accuracies ranging from 91.68% to 99.77% with no significant differences observed. Conversely, the user accuracy showed substantial fluctuations, varying from 26.58% to 94.05%, with FC 4 obtaining the lowest outcome and FC 15 achieving the highest, followed by FC 7, which recorded a satisfactory level of 93.94%. In terms of F1 scores, the range was from 38.42% to 96.67%, with FC 4 presenting the lowest value. The top five feature combinations with the highest F1 score were FC 7 (96.67%), FC 13 (95.14%), FC 12 (94.24%), FC 14 (93.43%), and FC 15 (92.75%); among these, FC 15 exhibited an increase of 2.71% when compared with the RGB image. In terms of composite score ranking (**Supplementary Table S1**), FC15 achieved the highest (0.91), followed by FC13 (0.89), FC14 (0.88), and FC12 (0.88), while FC7, despite having the highest F1 score (96.67%) and high UA, ranked fifth (0.86) due to its lower overall accuracy (72.53%).

When analyzing the SVM classifier results, we found that the producer accuracy fluctuated between 45.43% and 99.64%, with FC 4, FC 7, FC 3, FC 6, and FC 11 registering the lowest rates, which were 45.43%, 65.66%, 82.21%, 84.19, and 89.02%, respectively; their low composite scores (0.37–0.77) reflected this poor PA performance. Conversely, the user accuracy ranged from 32.7% to 92.68%, with FC 4 demonstrating the lowest outcome and FC 15 scoring the highest, followed by FC 14, which registered a satisfactory level of 92.91%. In terms of F1 scores, the range was observed to be between 37.78% and 95.97%, with FC 4 presenting the lowest value. The five feature combinations with the highest F1 score were FC 15 (95.97%), FC 14 (92.91%), FC 9 (91.92%), FC 12 (91.77%), and FC 10 (88.56%); among these, FC 15 increased by 6.63% compared with the RGB image. The composite scoring (**Supplementary Table S2**) confirmed FC15’s superiority (0.93) and showed that the top-ranked combinations (FC15 followed by FC14, FC9, FC12, and FC13, all with scores ≥0.85) aligned closely with the top F1 performers.

**Figure 8:**
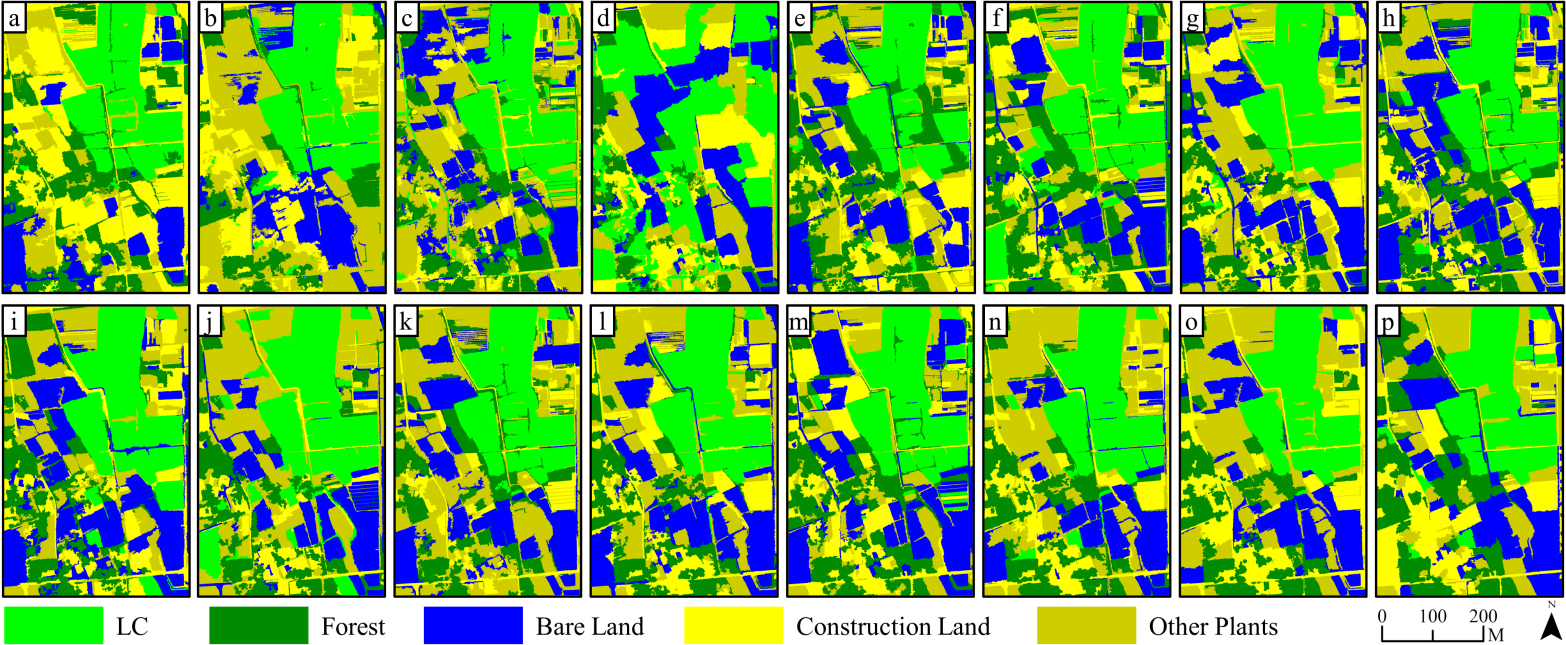
Results of KNN (object-oriented) classification on 15 kinds of feature combinations: **(a–o)** are classification results based on FC1~FC 15, while **(p)** is the classification results based on RGB image.

**Figure 9:**
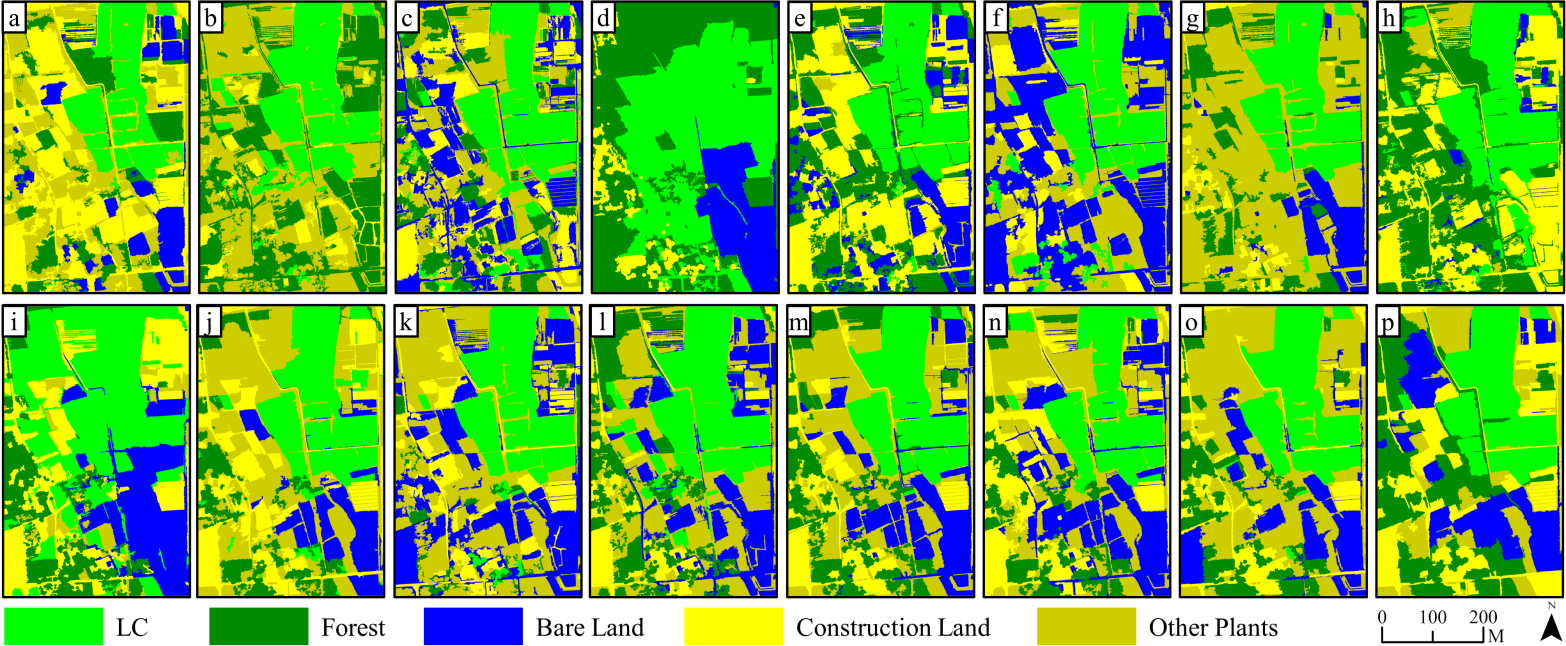
Results of SVM (object-oriented) classification on 15 kinds of feature combinations: **(a–o)** are classification results based on FC1~FC 15, while **(p)** is the classification results based on RGB image.

In comparison with the outcomes of pixel-based classification, **Figures 8** and **9** show that, as the number and diversity of features utilized in object-oriented classifications increased, the accuracy of land object classifications and the precision of LC cultivation area extractions displayed an upward tendency. Classifications involving four single feature variables found it difficult to effectively distinguish land objects. Specifically, a substantial number of errors occurred in the extraction of LC cultivation areas in FC 3 and FC 4, which relied on features such as texture and the digital surface model. Conversely, when two feature variables were combined in FC 5–10, the classification effect was significantly improved, the salt and pepper effect decreased, yet complete recognition of LC plots remained elusive. The classification outcomes for FC 11–14, which involved the combination of three feature variables, were further improved compared with FC 5–10. Notably, the misclassification and salt and pepper effects in the extraction results of the LC cultivation area in FC 13 were significantly improved compared with any other combinations. Finally, FC 15, which incorporated all four features, demonstrated the most effective result in land classification and LC cultivation area extraction, with complete recognition of all LC plots and only a minor number of other vegetation objects being misclassified as LC.

**Figure 10:**
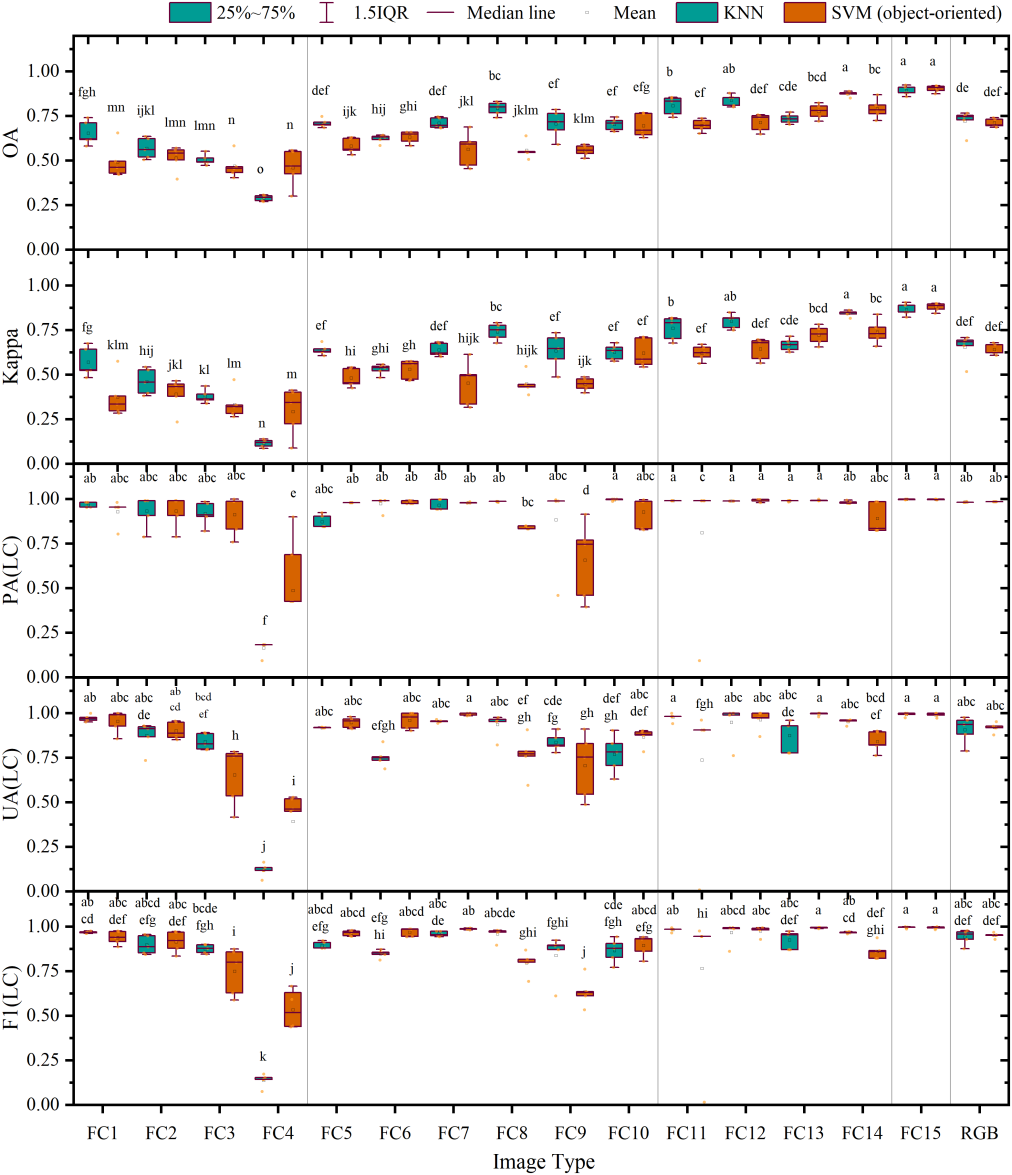
Accuracy evaluation of the results of object-oriented classification: the letters on the top of charts shows that the differences are significant.

As depicted in **Figure 10**, when classifying FC 1–4 based on a single feature variable, the average overall accuracies were all below 70.00%, and the average Kappa coefficients were all lower than 0.60, with correspondingly low composite scores (KNN: 0.17–0.75; SVM: 0.47–0.73), among which the average overall accuracy and average Kappa coefficient of FC 4 were the lowest and its composite scores ranked last in both classifiers (KNN: 0.17; SVM: 0.47). In FC 5–10 combined by two feature variables, except for FC 5, the average overall accuracies were all between 55.88% and 79.33%, while the average Kappa coefficients varied between 0.45 and 0.74, yielding composite scores of 0.68–0.88 (KNN) and 0.60–0.86 (SVM). When comparing FC 11 through 14 grouped by three feature variables, the average overall accuracies and average Kappa coefficients were further enhanced as compared with FC 5–10, respectively, between 69.85 and 87.52% and 0.64 and 0.86, achieving composite scores of 0.70–0.92 (KNN) and 0.62–0.90 (SVM). Here, the average overall accuracy and average Kappa coefficient of FC 14 (KNN) reached 87.52% and 0.84, respectively, with composite score of 0.92, second only to FC 15, which had four features. For this group, FC15 consistently achieved optimal composite scores across classifiers (KNN: 0.95; SVM: 0.95), with the average overall accuracy and average Kappa coefficient reached 89.16% (KNN), 90.36% (SVM), 0.86 (KNN), and 0.88 (SVM). When compared with the results of average overall accuracy and average Kappa coefficient based on a RGB image, FC 15 exhibited significant improvements of 17.03% (KNN), 19.20% (SVM), 0.21 (KNN), and 0.24 (SVM).

Similar to findings in pixel-based classification, our results from object-oriented classification demonstrated that in the context of LC cultivation plot extraction, the performance of the KNN classifier varied widely across feature combinations, ranging from 16.54% to 99.86% in terms of producer accuracy, with the sole exception of FC 4, whose accuracy was below 87.36%. The user accuracy ranged from 11.94% to 99.39%, with FC 4 registering the lowest result and FC 15 achieving the highest, followed by FC 11, which recorded 97.82%. The composite scoring (**Supplementary Table S3**) revealed FC15 as the top performer (0.95), with FC14 close behind at 0.92, despite FC11 having higher PA/UA values. In terms of F1 score, the top five feature combinations with the highest F1 score were FC 15 (99.62%), FC 11 (98.44%), FC 1 (96.87%), FC 12 (96.77%), and FC 14 (96.71%), though composite scoring prioritized FC15 (0.95), FC14 (0.92), FC12 (0.91), FC11 (0.90) and FC8 (0.88) due to balanced metric performance, with FC 4 again displaying the lowest score. Notably, FC 15 increased by 5.29% compared with the RGB image.

For the SVM classifier, we observed a producer accuracy range of 48.84% to 99.80%, with the vast majority of feature combinations demonstrating accuracy levels above 81.15% without significant differences. Nonetheless, the user accuracy varied from 39.27% to 99.54%, with FC 4 scoring the lowest and FC 7 and FC 13 recording the highest scores, closely followed by FC 15, which achieved 99.14%. Composite scoring (**Supplementary Table S4**) confirmed FC15’s dominance (0.95), with FC13 ranking second at 0.90, despite having lower OA than FC14. In terms of F1 score, the top five feature combinations with the highest scores were FC 15 (99.47%), FC 13 (99.46%), FC 7 (98.78%), FC 12 (97.69%), and FC 6 (97.01%), while composite scoring identified FC15 (0.95), FC13 (0.90), FC12 (0.86), RGB (0.84) and FC14 (0.82) as optimal, highlighting FC12’s strong overall performance. Interestingly, FC 15 displayed an increase in its F1 score of 4.31% compared with the RGB image results.

### 4.3 Method validation

**Figure 11:**
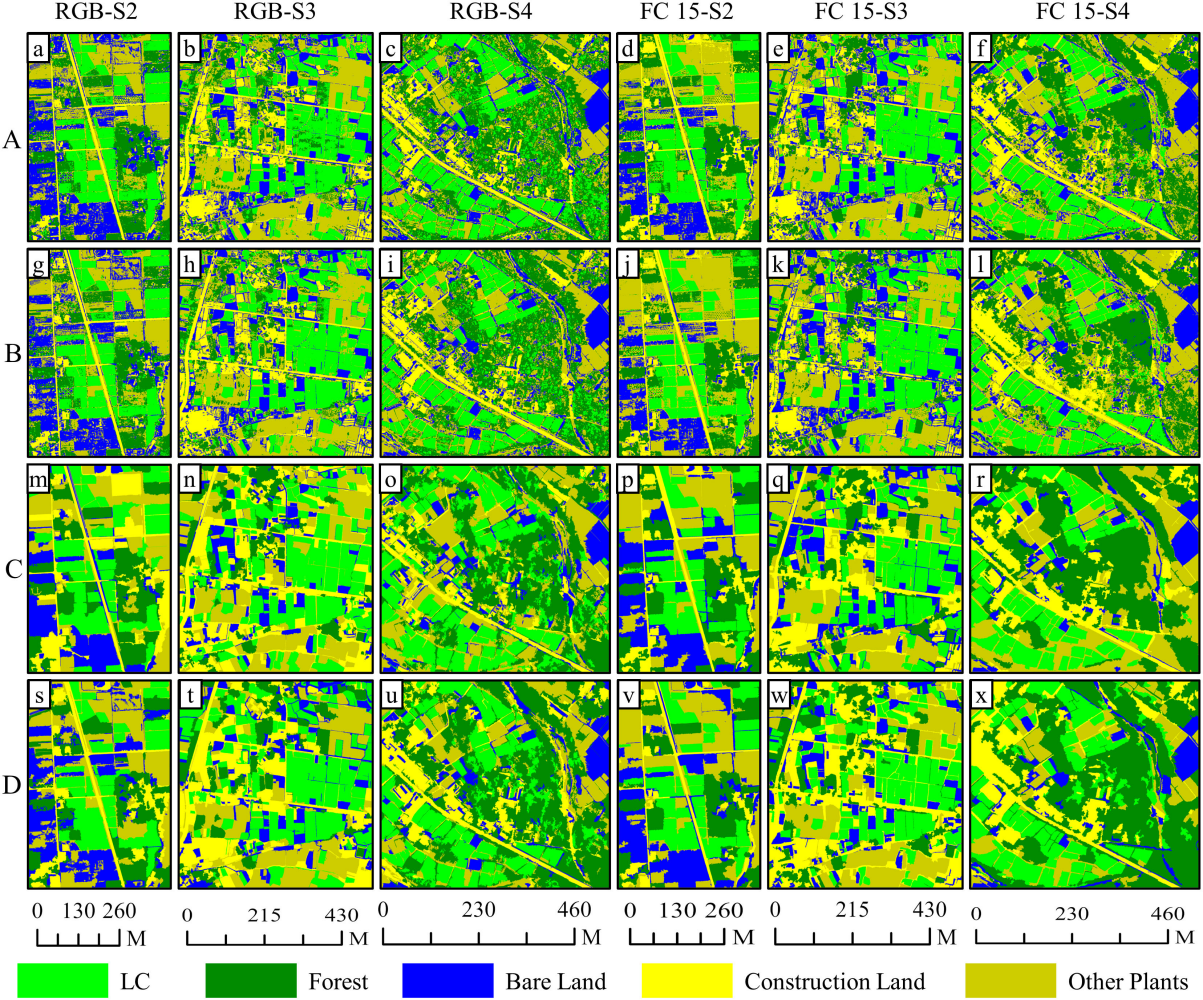
Comparison of classification results based on RGB images and feature variable combination images on S2, S3 and S4: **(A)** stands for the results of maximum likelihood classifier; **(B)** stands for the results of support vector machine classifier (pixel-based); **(C)** stands for the results of k-nearest neighbor classifier; **(D)** stands for the results of support vector machine classifier (object-oriented).

**Figure 12:**
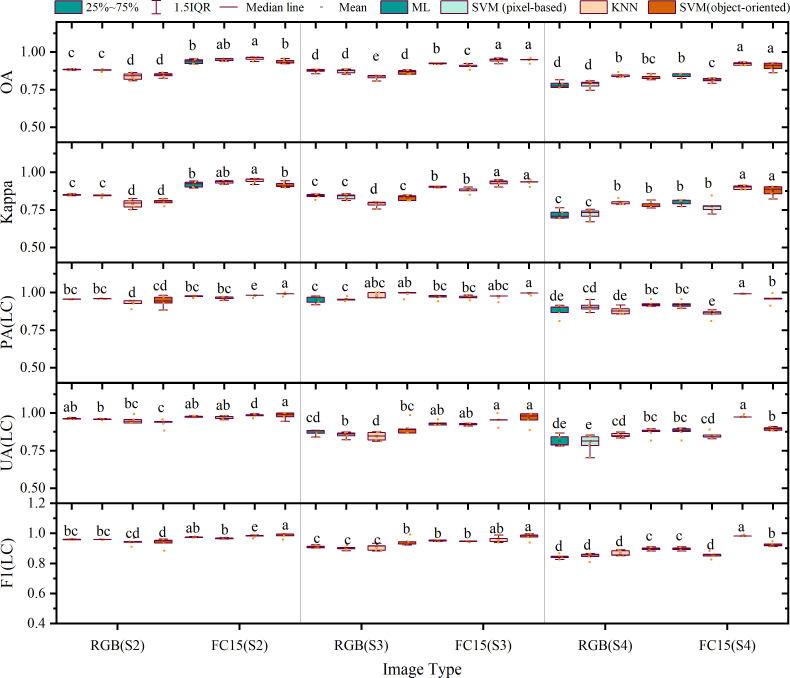
Accuracy evaluation of the results of pixel-based classification and object-oriented classification.

In **Figures 11** and **12**, we observed the classification outcomes of RGB images of S2, S3, and S4, along with their respective combinations of all four feature variables, using pixel-based and object-oriented classification approaches. In the pixel-based classifications, the RGB images and feature combination images demonstrated effective categorization of land objects. For instance, in the image in S2, the salt and pepper effect in the classification results based on feature combination images was the lightest, whereas there were numerous instances where forest pixels were incorrectly labeled as LC plot pixels within the RGB image classification. The accuracy of the four sets of image classification results in S3 was at a high level, but there were certain cases of missed classification. Finally, due to the late image acquisition time of images in S4, shadows dominated the visuals, leading to generally low classification accuracy. forest pixels and other plant pixels were sometimes misclassified as LC plot pixels, causing substantial salt and pepper effects.

On the contrary, in the object-oriented classifications, the presence of the salt and pepper effect was less pronounced in the RGB and feature variable combination classifications for S2. Within the RGB image classification for S2, there were cases where LC plots were misclassified as other plants due to inadequate growth and minimal vegetation coverage, whereas this plot was accurately classified when employing the feature variable combination approach. In the image classification results of S3, the extraction results of the LC cultivation area were relatively accurate. Finally, in the image classification results of S4, the classification accuracy based on feature combination images was significantly improved compared with pixel-based classification, accompanied by a decrease in the misclassification of forests as LC plots.

Specifically, as shown in **Figure 12**, in the outcome of pixel-based classifications, the average overall accuracies of classification based on RGB images from S2, S3, and S4 were 89.46%, 88.38% (ML), 87.96% (SVM), 87.53% (ML), 87.20% (SVM), and 78.20% (ML), 78.40% (SVM), respectively; the average Kappa coefficients were 0.85 (ML), 0.84 (SVM), 0.84 (ML), 0.84 (SVM), and 0.72 (ML), 0.72 (SVM). On the contrary, the average overall accuracies based on feature combination images were 93.77% (ML), 95.01% (SVM), 92.34% (ML), 90.66% (SVM), and 84.22% (ML), 81.40% (SVM), respectively, accompanied by Kappa coefficients of 0.92 (ML), 0.94 (SVM), 0.90 (ML), 0.88 (SVM), and 0.80 (ML), 0.77 (SVM), respectively. The use of feature combination images leads to statistically significant improvements in overall accuracy ranging from 5.39% (ML), 7.05% (SVM), 4.81% (ML), 3.46% (SVM), and 6.02% (ML) to 2.99% (SVM) for each method, along with improvements in the Kappa coefficient of 0.07 (ML), 0.09 (SVM), 0.06 (ML), 0.04 (SVM), and 0.08 (ML) to 0.05 (SVM) in the Kappa coefficient (p< 0.05).

In the outcome of object-oriented classification, average overall accuracies of classification based on RGB images from S2, S3, and S4 were 83.78% (KNN), 84.84% (SVM), 83.31% (KNN), 86.70% (SVM), and 84.69% (KNN), 83.43% (SVM), respectively; the Kappa coefficients were 0.79 (KNN), 0.80 (SVM), 0.79 (KNN), 0.83 (SVM), and 0.80 (KNN), 0.79 (SVM), respectively. Alternatively, when feature combination images were used, the overall accuracy reached 95.72% (KNN), 93.58% (SVM), 94.55% (KNN), 94.70% (SVM), and 92.25% (KNN), 90.35% (SVM), respectively, with respective Kappa coefficients of 0.94 (KNN), 0.92 (SVM), 0.93 (KNN), 0.93 (SVM), and 0.90 (KNN), 0.88 (SVM). The employment of feature combination images demonstrated statistically significant enhancements in overall accuracy between 11.95% (KNN), 8.74% (SVM), 11.24% (KNN), 8.00% (SVM), and 7.56% (KNN), 6.93% (SVM) in overall accuracy, while the Kappa coefficients improved by 0.15 (KNN), 0.11 (SVM), 0.14 (KNN), 0.10 (SVM), and 0.10 (KNN), 0.09 (SVM) (p< 0.05).

## 5 Discussion

### 5.1 Feasibility of extracting LC cultivation plots using feature variable combination

The spectral similarity among various crops and vegetation types presents a challenge for utilizing remote sensing technologies in detecting crop cultivation regions (Karmakar et al., 2024). Moreover, economic crops like LC are typically grown across scattered and fragmented plots (**Figure 11**), often with cluttered backgrounds, which contributes to their greater difficulty in identification compared to food crops. In our study, accurately classifying pixels of LC plots, forests, and other plants is a core issue. Consistent with the findings of Yang et al (Yang et al., 2022), we discovered that relying solely on RGB imagery makes it difficult to distinguish between vegetation types (**Figures 5**, **6**, **8**, **9**, and **11**). Consequently, incorporating appropriate feature variables to enhance classification accuracy becomes crucial. Since vegetation exhibits strong reflectivity in the near-infrared band and strong absorption in the red band, vegetation indices based on infrared wavelengths (e.g., NDVI) are widely used for extracting vegetation information (Liu et al., 2021). However, low-cost RGB cameras, lacking infrared bands, must utilize the strong reflectivity of vegetation in the green wavelength band and weak reflectivity in the red and blue wavelength bands to construct color indices such as GCC (Křížová et al., 2022).

Therefore, this study investigated the potential for synergistic enhancement in LC plot identification through multidimensional characterization enabled by combining diverse features. The mechanism by which the selected features facilitate extraction is analyzed as follows: VDVI builds on the computational methodology of the Normalized Difference Vegetation Index (NDVI) by integrating visible band data from RGB imagery to generate enhanced composite images. These synthesized outputs effectively enhance contrast between vegetated and non-vegetated areas (Zang et al., 2024). Building upon this foundation, Hue, distinguished by its strong robustness against illumination variations, preferentially differentiates vegetation areas from non-vegetated backgrounds (e.g., the brownish-yellow hue of bare land, non-green tones of construction land). Its ability to capture the unique chlorophyll reflectance characteristics of LC aids in the preliminary separation of other vegetation types (Majer et al., 2010; Lazarević et al., 2021). Concurrently, The discriminatory power of B-MEA stems from their robust correlation with the Leaf Area Index (LAI) (Xu et al., 2025). Manicured LC plots exhibit significantly greater texture homogeneity than natural vegetation in this spectral region due to their elevated vegetation coverage (corresponding to higher LAI), effectively enabling discrimination from other vegetation types. The introduction of DEM data further imposes vertical constraints. By synergizing with spectral-textural features, it enhances the separability between low-stature LC plots (~1m plant height) and forest while aiding in differentiating other vegetation types of comparable height. This multi-tiered discriminatory framework, integrating color attributes, spectral responses, textural structures, and topographic elevation, collectively forms the theoretical basis for distinguishing LC plots from four typical land cover types.

Fortunately, the shortcomings of spectral features are significantly improved after adding texture features, color space elements, and DEM data (**Figures 7**, **10**, and **12**). The discovery that different combinations of features lead to increased classification accuracy also validates the consistency of previous studies (Fu et al., 2021, 2022). Ultimately, the average overall accuracy of FC 15, calculated using all four machine learning algorithms—ML, SVMs (pixel-based), KNNs, and SVMs (object-oriented)—was 90.00%, 90.43%, 89.16%, and 90.36%, respectively. This result was significantly higher (by 14.26%–19.20%) than the classification accuracy achieved using only RGB images. The validation experiments executed on images captured from S2, S3, and S4 demonstrate that the feature variable combination images developed within this study exhibit consistent precision improvements across diverse regions. Notably, while the pixel-level classification precision of S4 images stands at 84.22% and 81.40%, respectively, the average overall accuracy of land object classification employing feature combination images surpasses 90.00%. Furthermore, except for the pixel classification precision of S4 images being recorded at 89.71% and 89.51%, the average F1 score of all other images transcends 90.00%. A comparison between pixel classification and object-oriented classification approaches divulges negligible differences in their precision for categorizing land objects; however, the latter technique demonstrates superior precision in detecting grassland farmland areas and greater steadiness in its performance.

### 5.2 Application of LC cultivation plot extraction

Traditional LC cultivation methods rely on manual ground inspections to assess the growth status and health of crops. Such methods are susceptible to observer subjectivity and experience, leading to diagnostic errors, especially when the cultivated area is expanding. Human labor alone struggles to meet the demand for timely and accurate inspections over large areas. In contrast, the use of a UAV greatly reduces inspection time—it takes only approximately 1 hour to complete an examination of 10 ha of land (typically involving far more than 100 LC cultivation plots). With the aid of the classification algorithm proposed in this article and the integration of image feature variables, it is possible to efficiently locate and identify all LC fields within only 2 hours. The resulting UAV images and videos can be safely stored on electronic media for future retrieval and analysis. Moreover, new algorithms can be easily applied to existing databases to reduce redundant data collection, thereby lowering cost inputs.

Based on the image information captured by RGB UAV in LC fields, we can preliminarily understand the planting overview of each block through the comparison of RGB colors and make simple judgments. Furthermore, we can precisely calculate the vegetation coverage rate of each plot using threshold segmentation or supervised classification methods to quantitatively analyze the density of the aboveground parts and their growth trends. Furthermore, analyzing images of LC plots from varying periods allows us to assess phenotypic indicators such as emergence uniformity, leaf area index, leaf chlorophyll and nitrogen content, aboveground biomass, and vegetation coverage under different fertilization and irrigation regimes. Indicators above usually precisely mirror the growth conditions of crop (Maimaitijiang et al., 2020; Wan et al., 2020; Zhang et al., 2024). Consequently, our objective is to formulate high-resolution, plot-level models for LC yield prediction and management strategy recommendations.

### 5.3 Limitations, challenges, and future steps

It is noteworthy that while exploring feature combinations (color indices, texture features, color spaces, DEM) significantly enhanced the accuracy of LC plot cultivation parcel extraction from UAV-based RGB imagery, inherent limitations of RGB data and methodological constraints remain core challenges requiring further attention.

#### 5.3.1 RGB data constraints

##### 5.3.1.1 Illumination sensitivity

The primary challenge lies in its extreme sensitivity to illumination variations. The significant accuracy declines in S4 (7.47% and 6.01% lower mean overall accuracy than S2 and S3, respectively) directly demonstrates the strong negative impact of shadows on classification outcomes. This area was imaged at 5:00 PM, generating extensive shadows within forested regions. Therefore, for practical implementation, image acquisition during periods of sufficient illumination and minimal shadows is strongly recommended to optimize accuracy.

##### 5.3.1.2 Seasonal variation

Furthermore, this study needs to further evaluate the potential influence of seasonal variations. Dynamic changes in canopy color, coverage, and background conditions (e.g., proportion of bare soil) of LC, other plants, and forest across phenological stages may significantly affect the stability of key discriminative features such as the VDVI and Hue. Future work should incorporate multi-temporal data spanning complete growing seasons to systematically assess seasonal robustness.

#### 5.3.2 Georeferencing and geometric constraints

Absolute positioning accuracy limitation: This study did not utilize Ground Control Points (GCPs) during data processing. The SfM 3D reconstruction relied solely on the GNSS positioning data from the UAV (DJI Mavic 2 Pro). Consequently, the absolute georeferencing accuracy of the final DOM and DEM products is inherently constrained to an estimated horizontal accuracy of 2–5 meters (Elkhrachy, 2021), with an associated risk of geometric distortions during processing. This limitation hinders the direct applicability of the classification results in scenarios demanding high geospatial precision, such as precise co-registration with high-accuracy base maps, sub-meter-level area calculation, or accurate multi-temporal change detection. However, all study sites (S1–S4) are situated within the gently undulating terrain of the Chengdu Plain, where minimal topographic relief significantly reduces the risk of elevation distortion artifacts in SfM-derived products. Visual inspection confirmed that the generated DOM/DEM effectively preserved the relative geometric relationships of key landscape features (e.g., field boundaries, roads). Critically, the classification methodology fundamentally exploits discriminative features—including spectral reflectance, textural patterns, color space components, and local topography (DEM)—between target LC plots and background features. The separability of these features is maintained as long as internal geometric consistency (i.e., absence of destructive warping of object outlines) is preserved within the DOM/DEM across the study area. Furthermore, training and validation samples were annotated directly on the DOM imagery, ensuring perfect spatial alignment between reference data and classification inputs. Therefore, evaluation metrics (overall accuracy, Kappa coefficient, F1-score) reflect the model’s performance within this consistent geometric framework, not its absolute geospatial positioning accuracy. Synthesizing these considerations, we contend that the geolocation accuracy limitation exerts a minimal impact on the study’s primary objective: identifying L. chuanxiong cultivation plots using UAV RGB-derived feature combinations.

#### 5.3.3 Challenges from high spatial resolution

##### 5.3.3.1 Intra-class variation & background heterogeneity

Another challenge amplified by high spatial resolution is intensified intra-class spectral variation and background heterogeneity. While centimeter-scale resolution greatly mitigates the mixed-pixel problem, it also accentuates spectral differences among identical objects (e.g., LC plots under varying health statuses or micro-environments) and highlights intricate background details like weeds, crop residues, and uneven soil moisture within plots. This complexity introduces additional noise for fine-grained classification.

##### 5.3.3.2 Processing efficiency

Moreover, the massive data volume from high resolution imposes higher demands on processing efficiency. Future studies need comprehensive evaluation of flight altitudes (corresponding to varying resolutions) to balance interlinked effects on spectral variation, classification accuracy, and computational efficiency.

#### 5.3.4 Feature selection strategy limitations

##### 5.3.4.1 Potential geographic bias

Regarding feature selection strategy, the identification of core features (VDVI, B-MEA, Hue) and the optimal combination FC15 relied solely on S1 data. Though validated across S2-S4, these region-specific results may exhibit geographical bias, and their generalizability warrants large-scale verification across broader geographic settings, soil types, and cultivation practices.

##### 5.3.4.2 Lack of quantifiable validation

Critically, the current interpretation of feature roles relies on speculative physiological reasoning (e.g., canopy chlorophyll differences) rather than quantifying their actual contribution to model decisions, limiting understanding of the model’s internal mechanisms and validation of feature selection efficacy.

To address these challenges, future research should focus on:

###### 5.3.4.2.1 Enhancing robustness to RGB & geometric limitations

Develop preprocessing techniques (e.g., shadow detection/correction) or feature extraction methods less sensitive to illumination/shadow; systematically study temporal feature dynamics across LC growing seasons; evaluate resolution trade-offs; explore fusion with complementary data (e.g., near-infrared, multi-temporal/multi-angle imagery) or employ deep learning models (e.g., CNN, Transformer) to automatically learn discriminative and noise-robust features. For applications requiring high geospatial precision (e.g., precision agriculture management, high-accuracy area statistics, fusion with multi-source high-precision GIS data), future studies should incorporate Ground Control Points (GCPs) with RTK-corrected references or utilize UAVs equipped with RTK/PPK positioning systems during data acquisition. This will significantly enhance georeferencing accuracy and the practical utility of the outputs. The feature combinations and classification methods proposed in this study hold greater application potential when combined with high-precision spatial data.

###### 5.3.4.2.2 Deepening feature understanding & optimizing feature engineering

Prioritize integrating eXplainable AI (XAI) methods (e.g., SHAP values, LIME, model-inherent feature importance) (Gevaert, 2022) to quantify the contribution magnitude and direction of each input feature (including VDVI, B-MEA, Hue, and potential alternatives). This provides data-driven, quantifiable validation for feature selection, clarifies feature roles, and may reveal novel critical information. Concurrently, expand the initial feature library by incorporating more diverse texture features (e.g., Tamura texture, autoregressive models, wavelet features (Humeau-Heurtier, 2019)) and color spaces (e.g., CMY, LAB, YUV (Tokarczyk et al., 2014; Kamiyama and Taguchi, 2017)). Employ systematic feature reduction and selection strategies, such as synthesizing all potential features into multi-band imagery followed by reverse analysis using Principal Component Analysis (PCA), Minimum Redundancy Maximum Relevance (mRMR), or Recursive Feature Elimination (RFE) (Ram et al., 2024) to identify more generalizable and discriminative combinations.

## 6 Conclusion

This study successfully achieved high extraction accuracy for LC cultivation plots utilizing cost-effective UAV-based RGB imagery, thus addressing a gap in the existing literature. Prior research has predominantly focused on identifying cultivation areas for food crops while paying minimal attention to the accurate extraction of medicinal plant cultivation plots employing UAVRS technologies. This study filled this gap by extracting color indices, textural features, color spatial components, and DEM from UAV-based RGB imagery. Unlike earlier studies that explored intricate processes for feature dimensionality reduction, which might potentially result in inadequate generalization performance, this study employed Pearson correlation coefficient analysis to assess multicollinearity issues among different variables. Subsequently, the study compared the interclass difference scores of diverse land cover types with LC cultivation plots under various feature variables to select the most promising features in three characteristics combined with DEM for feature combination mode screening. This approach not only simplified the feature selection process but also reduced the dimensions of the feature combination, effectively improving classification efficiency. The results demonstrated robust stability in validation experiments across different production regions, suggesting its potential for large-scale application. Accurate LC cultivation plot identification provides a strong foundation for effective LC management in Sichuan Province, significantly contributing to the progression of LC precision agriculture implementation levels. This technique is expected to play a crucial role in the execution of quantitative management strategies such as nutrient status monitoring, precision irrigation, and quantitative fertilization.

## Data Availability

The raw data supporting the conclusions of this article will be made available by the authors, without undue reservation.
