# Supplementary material for: Extracting Ligusticum chuanxiong Hort. cultivation plots based on feature variable combinations constructed from UAV-based RGB images
Source: Front Plant Sci. 2025 Nov 10;16:1659442. doi: 10.3389/fpls.2025.1659442 (PMC12640989; doi:10.3389/fpls.2025.1659442)
Supplement: Supplementary file 1 [file Table1.docx]

**Table S1**

Composite scores and rankings of feature combinations for pixel-based classification using maximum likelihood classifier (ML)

| Image Type | Classifier | OA | Kappa | PA | UA | F1 | Composite Score | Rank |
| --- | --- | --- | --- | --- | --- | --- | --- | --- |
| FC15 | ML | 90.00±3.11% | 0.87±0.04 | 92.49±9.46% | 94.05±7.07% | 92.75±3.78% | 0.91 | 1^*^ |
| FC13 | ML | 82.90±3.11% | 0.79±0.04 | 98.10±1.09% | 92.43±4.77% | 95.15±3.02% | 0.89 | 2^*^ |
| FC14 | ML | 82.71±1.76% | 0.78±0.02 | 99.03±1.13% | 88.46±1.89% | 93.44±0.96% | 0.88 | 3^*^ |
| FC12 | ML | 80.53±3.73% | 0.76±0.05 | 99.46±0.40% | 89.90±9.14% | 94.24±5.20% | 0.88 | 4 |
| FC7 | ML | 72.53±3.71% | 0.66±0.05 | 99.59±0.30% | 93.94±2.48% | 96.67±1.42% | 0.86 | 5 |
| FC10 | ML | 80.21±1.64% | 0.75±0.02 | 96.75±2.29% | 80.15±2.27% | 87.65±1.86% | 0.84 | 6 |
| RGB | ML | 75.74±2.01% | 0.69±0.02 | 93.79±2.42% | 86.76±4.13% | 90.04±1.41% | 0.83 | 7 |
| FC9 | ML | 73.54±1.11% | 0.67±0.01 | 99.16±1.34% | 82.92±2.10% | 90.31±1.67% | 0.83 | 8 |
| FC11 | ML | 72.33±2.68% | 0.68±0.06 | 91.68±6.44% | 80.77±5.26% | 85.63±2.66% | 0.80 | 9 |
| FC8 | ML | 71.12±1.18% | 0.64±0.01 | 96.95±2.43% | 73.36±3.69% | 83.43±1.62% | 0.78 | 10 |
| FC6 | ML | 62.33±2.31% | 0.53±0.03 | 97.49±3.74% | 75.45±5.53% | 84.87±2.23% | 0.74 | 11 |
| FC5 | ML | 60.11±2.14% | 0.50±0.03 | 99.41±0.44% | 66.56±4.49% | 79.66±3.08% | 0.71 | 12 |
| FC2 | ML | 61.10±1.09% | 0.51±0.01 | 99.11±1.39% | 56.10±1.28% | 71.63±0.84% | 0.67 | 13 |
| FC1 | ML | 49.67±5.27% | 0.37±0.06 | 99.77±0.33% | 58.11±5.06% | 73.33±3.87% | 0.63 | 14 |
| FC3 | ML | 47.02±2.46% | 0.34±0.03 | 94.52±8.19% | 55.79±9.80% | 69.30±5.92% | 0.60 | 15 |
| FC4 | ML | 41.21±1.80% | 0.26±0.02 | 69.67±9.83% | 26.58±1.88% | 38.42±3.34% | 0.39 | 16 |

*p-value from Tukey HSD test; *p<0.05

**Table S2**

Composite scores and rankings of feature combinations for pixel-based classification using support vector machine classifier (SVM)

| Image Type | Classifier | OA | Kappa | PA | UA | F1 | Composite Score | Rank |
| --- | --- | --- | --- | --- | --- | --- | --- | --- |
| FC15 | SVM | 90.43±1.81% | 0.88±0.02 | 99.53±0.19% | 92.68±1.52% | 95.97±0.79% | 0.93 | 1^*^ |
| FC14 | SVM | 83.07±1.20% | 0.79±0.02 | 99.46±0.22% | 87.17±0.24% | 92.91±0.08% | 0.88 | 2^*^ |
| FC9 | SVM | 77.97±1.16% | 0.72±0.01 | 99.61±0.27% | 85.34±0.40% | 91.92±0.22% | 0.85 | 3^*^ |
| FC12 | SVM | 77.70±1.56% | 0.72±0.02 | 99.64±0.30% | 85.06±0.46% | 91.77±0.30% | 0.85 | 4^*^ |
| FC13 | SVM | 80.65±1.23% | 0.76±0.02 | 97.84±1.55% | 81.32±0.57% | 88.81±0.45% | 0.85 | 5^*^ |
| FC10 | SVM | 79.95±1.30% | 0.75±0.02 | 98.10±1.50% | 81.22±0.70% | 88.86±0.38% | 0.85 | 6^*^ |
| RGB | SVM | 72.99±2.91% | 0.66±0.04 | 93.53±3.02% | 85.76±4.74% | 89.34±1.51% | 0.82 | 7 |
| FC8 | SVM | 73.33±0.61% | 0.66±0.01 | 89.51±4.29% | 76.39±2.07% | 82.34±0.75% | 0.78 | 8 |
| FC11 | SVM | 73.07±0.61% | 0.66±0.01 | 89.02±3.12% | 75.96±1.52% | 81.93±0.47% | 0.77 | 9 |
| FC1 | SVM | 61.53±1.98% | 0.52±0.02 | 94.94±2.21% | 82.42±5.72% | 88.09±2.51% | 0.76 | 10 |
| FC2 | SVM | 66.05±2.81% | 0.57±0.03 | 95.30±2.61% | 66.43±5.27% | 78.12±3.13% | 0.72 | 11 |
| FC5 | SVM | 66.29±1.16% | 0.58±0.01 | 97.51±2.02% | 61.86±2.38% | 75.65±1.18% | 0.71 | 12 |
| FC6 | SVM | 62.00±1.37% | 0.52±0.02 | 84.19±4.43% | 64.75±1.96% | 73.11±0.87% | 0.67 | 13 |
| FC3 | SVM | 56.71±3.26% | 0.46±0.04 | 82.21±2.59% | 64.05±2.88% | 71.94±1.69% | 0.64 | 14 |
| FC7 | SVM | 45.64±8.10% | 0.31±0.10 | 65.66±26.37% | 38.42±7.31% | 47.08±14.31% | 0.45 | 15 |
| FC4 | SVM | 42.65±5.03% | 0.28±0.06 | 45.43±12.46% | 34.70±11.11% | 37.78±5.80% | 0.37 | 16 |

*p-value from Tukey HSD test; *p<0.05

**Table S3**

Composite scores and rankings of feature combinations for object-oriented classification using k-nearest neighbor classifier (KNN)

| Image Type | Classifier | OA | Kappa | PA | UA | F1 | Composite Score | Rank |
| --- | --- | --- | --- | --- | --- | --- | --- | --- |
| FC15 | KNN | 89.16±2.63% | 0.86±0.03 | 99.86±0.18% | 99.39±1.04% | 99.62±0.49% | 0.95 | 1^*^ |
| FC14 | KNN | 87.52±1.39% | 0.84±0.02 | 98.17±0.82% | 95.32±1.48% | 96.71±0.49% | 0.92 | 2^*^ |
| FC12 | KNN | 83.99±3.33% | 0.80±0.04 | 99.05±0.00% | 95.07±10.47% | 96.77±5.89% | 0.91 | 3 |
| FC11 | KNN | 80.91±5.19% | 0.76±0.07 | 99.09±0.00% | 97.82±2.09% | 98.44±1.07% | 0.90 | 4 |
| FC8 | KNN | 79.33±3.75% | 0.74±0.05 | 98.63±0.20% | 93.72±6.40% | 96.02±3.49% | 0.88 | 5 |
| FC7 | KNN | 71.11±3.05% | 0.64±0.04 | 96.58±2.97% | 95.52±0.82% | 96.03±1.47% | 0.85 | 6 |
| RGB | KNN | 72.12±6.25% | 0.65±0.08 | 98.36±0.29% | 90.90±7.60% | 94.34±4.13% | 0.84 | 7 |
| FC13 | KNN | 73.51±2.76% | 0.67±0.04 | 99.01±0.38% | 87.52±9.03% | 92.71±5.11% | 0.84 | 8 |
| FC1 | KNN | 65.49±6.80% | 0.57±0.08 | 96.61±1.50% | 97.17±1.81% | 96.87±0.59% | 0.83 | 9 |
| FC5 | KNN | 71.07±2.39% | 0.64±0.03 | 87.36±3.76% | 91.97±0.14% | 89.57±2.02% | 0.81 | 10 |
| FC10 | KNN | 70.36±3.46% | 0.63±0.04 | 99.69±0.50% | 77.09±10.62% | 86.61±6.74% | 0.79 | 11 |
| FC9 | KNN | 70.70±7.84% | 0.63±0.10 | 88.52±23.72% | 83.77±5.12% | 83.99±12.86% | 0.78 | 12 |
| FC2 | KNN | 56.93±5.93% | 0.46±0.07 | 93.40±8.93% | 87.47±8.13% | 89.87±5.29% | 0.75 | 13 |
| FC6 | KNN | 62.33±2.31% | 0.53±0.03 | 97.49±3.74% | 75.45±5.53% | 84.87±2.23% | 0.74 | 14 |
| FC3 | KNN | 50.49±3.05% | 0.38±0.04 | 91.91±6.60% | 84.05±4.59% | 87.57±2.48% | 0.70 | 15 |
| FC4 | KNN | 29.06±1.68% | 0.11±0.02 | 16.54±3.97% | 11.94±3.67% | 13.80±3.69% | 0.17 | 16 |

*p-value from Tukey HSD test; *p<0.05

**Table S4**

Composite scores and rankings of feature combinations for object-oriented classification using support vector machine classifier (SVM)

| Image Type | Classifier | OA | Kappa | PA | UA | F1 | Composite Score | Rank |
| --- | --- | --- | --- | --- | --- | --- | --- | --- |
| FC15 | SVM | 90.36±1.86% | 0.88±0.02 | 99.80±0.18% | 99.14±1.09% | 99.47±0.60% | 0.95 | 1^*^ |
| FC13 | SVM | 77.66±4.22% | 0.72±0.05 | 99.38±0.36% | 99.54±0.86% | 99.46±0.28% | 0.90 | 2^*^ |
| FC12 | SVM | 71.49±5.03% | 0.64±0.06 | 99.19±0.70% | 96.39±5.37% | 97.69±2.66% | 0.86 | 3 |
| RGB | SVM | 71.16±2.42% | 0.64±0.03 | 98.57±0.05% | 92.00±2.65% | 95.16±1.40% | 0.84 | 4 |
| FC14 | SVM | 79.10±5.42% | 0.74±0.07 | 89.14±8.72% | 84.11±5.80% | 86.27±4.74% | 0.82 | 5 |
| FC6 | SVM | 63.19±3.46% | 0.53±0.06 | 98.17±0.94% | 95.99±4.65% | 97.01±2.28% | 0.81 | 6 |
| FC10 | SVM | 69.56±6.69% | 0.62±0.08 | 92.72±8.75% | 86.81±4.72% | 89.56±5.91% | 0.80 | 7 |
| FC7 | SVM | 56.33±9.77% | 0.45±0.12 | 98.03±0.30% | 99.54±0.64% | 98.78±0.39% | 0.80 | 8 |
| FC5 | SVM | 58.23±4.36% | 0.48±0.05 | 98.08±0.09% | 94.81±3.00% | 96.40±1.55% | 0.79 | 9 |
| FC1 | SVM | 49.31±9.57% | 0.37±0.12 | 93.06±7.13% | 95.44±6.13% | 93.95±3.77% | 0.74 | 10 |
| FC2 | SVM | 51.44±7.08% | 0.39±0.09 | 93.40±8.93% | 90.22±4.85% | 91.63±5.96% | 0.73 | 11 |
| FC11 | SVM | 69.85±3.44% | 0.62±0.04 | 81.15±40.12% | 73.80±40.82% | 76.66±41.95% | 0.73 | 12 |
| FC8 | SVM | 55.88±4.84% | 0.45±0.06 | 84.03±0.85% | 76.44±11.16% | 79.71±6.36% | 0.68 | 13 |
| FC3 | SVM | 46.87±6.83% | 0.33±0.08 | 91.35±11.10% | 65.46±16.75% | 75.08±13.29% | 0.62 | 14 |
| FC9 | SVM | 55.68±3.18% | 0.45±0.04 | 65.78±22.09% | 70.61±18.28% | 63.54±8.18% | 0.60 | 15 |
| FC4 | SVM | 46.13±10.52% | 0.29±0.14 | 58.64±20.59% | 49.09±3.49% | 52.65±10.03% | 0.47 | 16 |

*p-value from Tukey HSD test; *p<0.05
